# Plasma Membrane Epichaperome–Lipid Interface: Regulating Dynamics and Trafficking

**DOI:** 10.3390/cells14201582

**Published:** 2025-10-11

**Authors:** Haneef Ahmed Amissah, Ruslana Likhomanova, Gabriel Opoku, Tawfeek Ahmed Amissah, Zsolt Balogi, Zsolt Török, László Vigh, Stephanie E. Combs, Maxim Shevtsov

**Affiliations:** 1 Department of Medical Biology and Biotechnology, Institute of Life Sciences and Biomedicine, FEFU Campus, Far Eastern Federal University, 690922 Vladivostok, Russia; haneefamissah@gmail.com; 2 Diagnostics Laboratory Department, Trauma and Specialist Hospital, P.O. Box 326, Winneba CE-122-2486, Central Region, Ghana; 3 Laboratory of Biomedical Nanotechnologies, Institute of Cytology of the Russian Academy of Sciences (RAS), 194064 Saint Petersburg, Russia; tagaeva97@yandex.ru; 4 Department of Medical Technology, Graduate School of Health Sciences, Okayama University, Okayama 700-8558, Japan; gabbyjnr53@gmail.com; 5 Department of Medical Laboratory Science, Faculty of Health and Allied Sciences, Koforidua Technical University, Koforidua EN-112-3991, Eastern Region, Ghana; tawfeek.amissah@ktu.edu.gh; 6 Institute of Biochemistry and Medical Chemistry, Medical School, University of Pécs, 7624 Pécs, Hungary; 7 Institute of Biochemistry, HUN-REN Biological Research Centre, 6726 Szeged, Hungary; ztorok@lipidart.com (Z.T.); vigh@lipidart.com (L.V.); 8 Department of Radiation Oncology, Technical University of Munich (TUM), Klinikum rechts der Isar, 81675 Munich, Germany; stephanie.combs@tum.de

**Keywords:** membrane-bound heat shock proteins, epichaperome, lipidomics, plasma membrane, heat shock proteins, membrane lipids

## Abstract

The plasma membrane (PM) of eukaryotic cells plays a key role in the response to stress, acting as the first line of defense against environmental changes and protecting cells against intracellular perturbations. In this work, we explore how membrane-bound chaperones and membrane lipid domains work together to shape plasma membrane properties—a partnership we refer to as the “epichaperome–plasma membrane lipid axis.” This axis influences membrane fluidity, curvature, and domain organization, which in turn shapes the spatial and temporal modulation of signaling platforms and pathways essential for maintaining cellular integrity and homeostasis. Changes in PM fluidity can modulate the activity of ion channels, such as transient receptor potential (TRP) channels. These changes also affect processes such as endocytosis and mechanical signal transduction. The PM proteome undergoes rapid changes in response to membrane perturbations. Among these changes, the expression of heat shock proteins (HSPs) and their accumulation at the PM are essential mediators in regulating the physical state and functional properties of the membrane. Because of the pivotal role in stress adaptation, HSPs influence a wide range of cellular processes, which we grouped into three main categories: (i) mechanistic insights, differentiating in vitro (liposome, reconstituted membrane systems) and in vivo evidence for HSP-PM recruitment; (ii) functional outputs, spanning how ion channels are affected, changes in membrane fluidity, transcytosis, and the process of endocytosis and exosome release; and (iii) pathological effects, focusing on how rewired lipid–chaperone crosstalk in cancer drives resistance to drugs through altered membrane composition and signaling. Finally, we highlight Membrane Lipid Therapy (MLT) strategies, such as nanocarriers targeting specific PM compartments or small molecules that inhibit HSP recruitment, as promising approaches to modulate the functional stability of epichaperome assembly and membrane functionality, with profound implications for tumorigenesis.

## 1. Introduction

The complexity of the plasma membrane (PM) architecture and lipidome governs its biophysical and signaling competencies. Lipidomic studies have revealed that these lipids, including glycerophospholipids/phospholipids, sterols, sphingolipids, and glycolipids, are not randomly distributed but instead interact in fluid lateral segregations, forming specialized microdomains that enable localized signal transduction and trafficking [1]. Large-scale molecular simulations on multiple length and time scales have revealed liquid-ordered and disordered phases, a moderately cholesterol-enriched exomembrane, and nanodomains of gangliosides (glycolipids containing sialic acids) and phosphatidylinositol bisphosphate/triphosphates (PIPs) [2,3] that influence the biophysical properties of the membrane, such as thickness and stiffness [4]. This dynamic yet strictly maintained composition is crucial for cellular adaptation and proper cell function.

In healthy cells, this architecture translates to precise control. Phospholipid asymmetry is maintained, with phosphatidylserine (PS) predominantly located in the inner leaflet of the membrane. This configuration facilitates efficient signaling and triggers apoptosis only when PS flips to the outer leaflet. Cholesterol levels are rigorously maintained to optimize membrane fluidity and raft organization, which are crucial for receptor localization and signaling. Sphingolipid metabolism promotes regulated growth and differentiation, thereby preventing aberrant cell behavior. Similarly, high PM plasticity, which is responsive to external stimuli, metabolites, and substrates, ensures flexible adaptation. Orchestrating this homeostatic equilibrium is the precise regulation of lipid enzyme activity and transporter function [1,5,6] that, through meticulous lipidome membrane translocation and assemblage, actively maintains cellular homeostasis.

**Figure 1:**
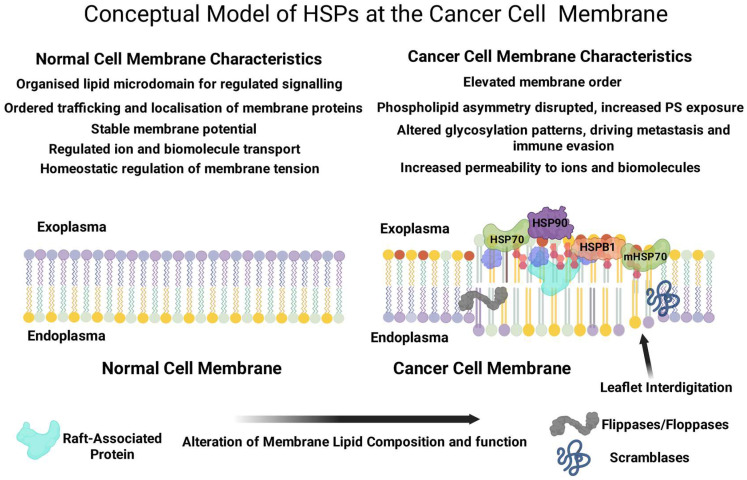
The figure compares plasma membrane lipids in healthy and cancer cells, highlighting the roles of altered lipid composition, HSP involvement, and membrane organization. Healthy cells feature organized lipid raft microdomains and symmetrical phospholipid distributions. In contrast, cancer cells exhibit disrupted rafts and asymmetrical distribution. HSP expression and localization also differ, with elevated exoplasmic binding and increased membrane-bound HSP70 (mHSP70) in cancer cells, indicating a potential therapeutic target.

In the transformed cell state, cancer cells hijack the PM, organizing aberrant lipid remodeling that undermines cellular order and promotes uncontrolled growth, invasion, and metastasis (Figure 1) [7]. Lipidomic profiling has revealed significant alterations in lipid signatures across diverse cancer types and grades [8,9,10,11]. This abnormal remodeling manifests in several key ways: The dysregulation of the phospholipid asymmetry, specifically PS externalization, promotes immune evasion and angiogenesis [12,13,14]. It is evident that PTEN loss and PI3K/Akt activation drive cholesterol ester accumulation and cancer aggressiveness. Plasma membrane biophysical activity (fluidity and stiffness) depends on free cholesterol content and leaflet symmetry. Cholesterol efflux in tumor associated macrophages (TAMs) reprograms TAMs and promotes tumor progression [15,16,17,18]. Furthermore, adipocytes rewire cancer cell metabolism by diverting glucose to glycerol-3-phosphate, augmenting PM glycerophospholipid biosynthesis and accumulation, which is crucial for metastatic behavior [19,20]. Notably, Dobrzyńska et al. [21] reported a characteristic increase in the PC/PE ratio in metastasizing malignant cells compared to non-metastasizing malignant cells, with a total positive charge at low pH and an increased total negative charge at high pH. Perturbed sphingolipid metabolism, with increased sphingosine-1-phosphate (S1P), stimulates cellular hyperplasia, angiogenesis, metastasis, and drug resistance [22]. A trade-off between ceramide and sphingomyelin enhances cancer cell resistance levels against apoptosis, promotes proliferation, and induces drug resistance. Equally, the enhanced activity of lipid translocases (flippases, scramblases, and floppases) drives accelerated lipid redistribution and PM remodeling, promoting incipient cancer phenotypes [23,24]. A collaborative marker of this process is the upregulation of *LPCAT1*. This enzyme is a key player in lipid remodeling, actively contributing to the development of distinct properties and composition of emergent cancer cell membranes [8]. Furthermore, increased sphingolipid levels can trigger the intracellular complement system, driving inflammasome-mediated metastasis [7].

Consequently, membrane-controlled processes, such as ion channel and action potential dynamics, exocytosis, endocytosis, and endo/exosomal pathways, are disrupted owing to changes in the stoichiometric properties of the membrane. This remodeling also affects the activity and clustering of PM-associated proteins. These drastic PM changes rewire cell signaling, subverting normal cellular control mechanisms and fueling the progression of cancer. Accumulating evidence implicates the activity of PM proteins in this process.

We propose the term “epichaperome–plasma membrane lipid axis” for a putative, interactive assembly of HSP70/HSP90 and co-chaperones (e.g., FKBP52, HOP, p23), scaffolded by anionic and raft lipids such as PS, cholesterol, and sphingomyelin. The components of this model rest on model-membrane and cellular studies but require targeted in vivo validation.

This axis integrates inputs from metabolic imbalances, hypoxia/ROS, and proteotoxic shock to yield measurable outputs, such as enhanced raft order, PS clustering, ion channel activity, and trafficking flux. The model predicts that stress-driven epichaperome assembly imposes order on disordered membrane domains and stabilizes the PM both directly and indirectly. By interacting with cytoplasmic and compartmental epichaperomes, it prolongs the signaling protein dwell time during sustained stress, thereby maintaining malignant phenotypes.

Here, we present an in-depth review of how HSPs interact with PM lipids, highlighting what is well understood and what remains unclear, and share actionable recommendations while highlighting crucial areas for future investigations and novel therapeutic discoveries.

## 2. Biophysical Impact of Lipid Membrane and Epichaperome Interactions

The lipid membrane is a multifactorial dynamic system that plays an indispensable role in maintaining cellular homeostasis and viability. For a long time, the plasma membrane was considered a simple matrix for embedded proteins, serving solely as a barrier with a strictly deterministic composition [25]. However, advances in physicochemical and molecular approaches have revealed the high heterogeneity, plasticity, and variability of the membrane. The cell membrane is involved in extracellular and intracellular transport, maintenance of optimal pH, cell signaling, communication, and interaction with the extracellular matrix. The structural diversity of lipids and, consequently, the complex composition of the membrane, which is regulated by an extensive group of lipid biosynthesis enzymes, determine its functional properties.

A critical feature of the cell membrane is lateral asymmetry, which promotes the clustering of specific lipid groups and the formation of regions differing in their phase state [25,26]. Liquid and solid, ordered and disordered lipid phases exhibit fundamentally distinct chemistry, biophysics, and thermodynamics; they differ in parameters such as thickness, rigidity, packing density, fluidity, and viscosity. Moreover, domains with varying properties and sizes can form both within the bilayer and in the monolayers of the same membrane region [27]. Such variable domain organization meets the local needs of the cell, enabling rapid and precise responses to external and internal stimuli of the cell.

The coexistence of phase-separated lipid domains in the membrane also favors the selective distribution of these proteins. The selectivity of protein molecules is determined not only by the chemical nature of the lipids but also by their preference for a specific phase state, which influences the geometry of functional protein domains. For example, many proteins are specific to lipid rafts—ordered, rigid membrane regions that serve as signaling platforms [28]. Changes in the physicochemical properties of rafts alter the affinity of certain proteins and their resulting cellular responses. For instance, the mechanism of action of some antitumor drugs is based on modulating apoptosis by recruiting and aggregating death receptors (Fas/CD95) in lipid rafts due to a local increase in ceramide levels [29,30]. The functioning and localization of GPI-anchored proteins, various receptors, channels, and small GTPases also depend on the characteristics of lipid rafts [28,31].

Model-membrane studies provide striking examples of chaperone selectivity towards specific phase states, importantly, the affinity of Hsp70 for saturated, PS-enriched ordered phases. These mechanistic data indicate plausible binding modes that require confirmation in native PM contexts. In a supported dipalmitoyl phosphatidylserine/dipalmitoyl phosphatidylcholine (DPPS/DOPC) bilayer assay, Lamprecht et al. [32] demonstrated using atomic force microscopy that purified HSP70 preferentially binds the saturated, negatively charged DPPS-rich ordered phase and forms stable protein clusters within this domain. Of note, when cholesterol was incorporated into the bilayer, HSP70 partially integrated into the membrane, resulting in increased membrane density [33]. This suggests that initial chaperone-membrane binding requires electrostatic interactions, whereas stable anchoring occurs via the alignment of protein domains with DPPS acyl chains. A similar mechanism has been proposed for regulating various lipid-protein interactions in ordered membrane domains [34]. An interesting observation was made by Mahalka et al. [35], who showed that HSP70 binds to liposomes containing either PS, cardiolipin, or bis-monoacylglycerophosphate in lysosomes via two tryptophan residues (W90 and W580) located in the N-terminal nucleotide-binding domain and C-terminal substrate-binding domain, respectively. The protein domains peripherally interacted with negatively charged lipids, altering their conformation and anchoring into the membrane via the insertion of lipid acyl chains into exposed hydrophobic cavities of HSP70. This confirms the chaperone’s specificity for both the lipid headgroup type and its fatty acid tail. These findings align with other studies showing that HSP70’s specificity for PS increases with the saturation of lipid acyl chains [36]. The presence of saturated fatty acid residues, which determine system order, is likely a prerequisite for proper chaperone conformation. Moreover, not only mammalian HSP70 but also its bacterial and yeast homologs exhibit specificity for marker lipids of detergent-resistant domains, such as globotriaosylceramide (Gb3), sulfogalactosylceramide (SGC), and sulfogalactosylglycerolipid (SGG) [37,38]. The essential cellular protein HSP90 binds to cholesterol, a stabilizing component of lipid rafts [39,40].

Further supporting chaperone preference for the physical state of the lipid membrane are proteomic studies detecting members of various HSP families in lipid rafts of tumor cells [41]. It is hypothesized that they form a stable cluster, allowing functional redundancy and maintenance of a pro-tumor phenotype even upon targeted inhibition of a single family member. Dores-Silva et al. showed that Hsc70/HSPA8 binds to anionic phospholipids and can possibly recruit HSP90 to PS-rich domains in model membranes, thereby functioning as a membrane-anchoring adaptor rather than a classical protein-folding chaperone [42]. In this context, the term “membrane-anchoring adaptor” specifically refers to the ability of HSP70 to stabilize lipid microdomains and scaffold additional chaperones at the bilayer interface. Consistent with this, lipidomic analyses of exosomes from PC-3 prostate cancer cells revealed that PS clustering and sphingomyelin species [43,44]. Biophysical studies further indicate that clustering of PS, particularly PS 18:0/18:1, can modulate the recruitment and activity of PS-binding proteins [45]. A fully functional membrane epichaperome may be essential for tumor cells to sustain functions such as motility, extracellular matrix association, or molecular transport across the lipid bilayer. These findings highlight the unique nature of chaperone–lipid membrane interactions, involving specific binding mechanisms to ordered phases and fine-tuned functional regulation.

Beyond exhibiting phase preference, some proteins can directly impact membrane biophysics, altering its domain organization. Such proteins are often termed membrane-active [46]. For instance, the neuronal tight junction protein NAP-22 promotes the formation of morphologically altered cholesterol-rich domains [47]. Similarly, mitochondrial creatine kinase and cytochrome c facilitate cardiolipin segregation and clustering, critical for apoptosis and energy metabolism [48]. The stress-inducible HSP12 of *Saccharomyces cerevisiae* also displays membrane-active properties. Welker et al. reconstituted yeast HSP12 in the negatively charged phosphatidylglycerol-containing membrane of liposomes and found it induces an intermediate ripple phase in vitro [49]. *Synechocystis* HSP17 exhibits specificity for anionic lipids and partially embeds in the hydrophobic bilayer core of the model membrane, promoting membrane ordering [50]. This property suggests a potential role in membrane thermostabilization, including in thylakoids. The bacterial chaperonin GroEL, a homolog of eukaryotic HSP60, increases membrane order (reducing fluidity and increasing rigidity) upon interaction with lipid bilayers of varying composition and thickness [51]. HSP27 and HSP90 alter lipid rotational and lateral mobility, modulating domain segregation and stabilizing membranes under stress [52,53,54]. Table 1 summarizes and categorizes the current understanding of the interaction of chaperones (HSPs) with PM lipids.

**Methodological caveats and recommended controls**. Some methods have specific artifacts: Detergent-resistant membrane (DRM) isolations can misrepresent native domains; methyl-β-cyclodextrin can perturb membranes more than cholesterol removal; liposomes and supported bilayer reconstitutions lack leaflet asymmetry and cytoskeletal context; AFM and some fluorescent probes can produce membrane surface/probe artifacts; and antibody staining and overexpression can mislocalize proteins. **Recommended orthogonal validation**: detergent-free fractionation or proximity-labeling for domain mapping; genetic or enzymatic cholesterol modulation with repletion rescue for cyclodextrin studies; live-cell LAURDAN phasor/TIR-FCS and single-molecule tracking could be useful to confirm model-membrane findings; endogenous tagging of acute targeted degradation rather than sole overexpression; and proximity-labeling lipidomics (PL-LipID/photo-crosslink) to identify direct lipid partners. (Authors should report lipid extraction protocols and detergent concentrations and, where possible, should include rescue experiments such as lipid repletion or non-binding/chaperone mutant controls).

Recently, our group demonstrated that HSP70 interaction with giant unilamellar PS-containing liposomes increases the area of solid ordered membrane domains, likely by reducing hydrophobic mismatch between domains via interdigitation induction [54]. Interdigitation is the reorganization of lipid molecules wherein acyl chains from opposing bilayer leaflets interpenetrate, reducing membrane thickness, increasing hydrophobic core pressure, altering surface electrostatics, and enhancing interleaflet coupling [43]. This mechanism may enable HSP70 to expand raft platforms for signaling modulation or facilitate ion-permeable pore formation by adapting the bilayer to its hydrophobic regions.

Presumably, increased lipid order and interdigitation modulation significantly influence adjacent protein functionality and cellular behavior by altering local membrane biophysics. Enhanced bilayer rigidity and reduced permeability directly affect cancer cell susceptibility to radio- and chemotherapy, signal cascade initiation, intracellular signaling, and molecular transport. For example, interdigitation between inner-leaflet stearoyloleoyl phosphatidylserine and outer-leaflet long-chain sphingomyelins promotes PS clustering, modulating PS-binding proteins (K-Ras, Akt, PKC, caveolins, Rho GTPases, etc.) [44,45].

**Figure 2:**
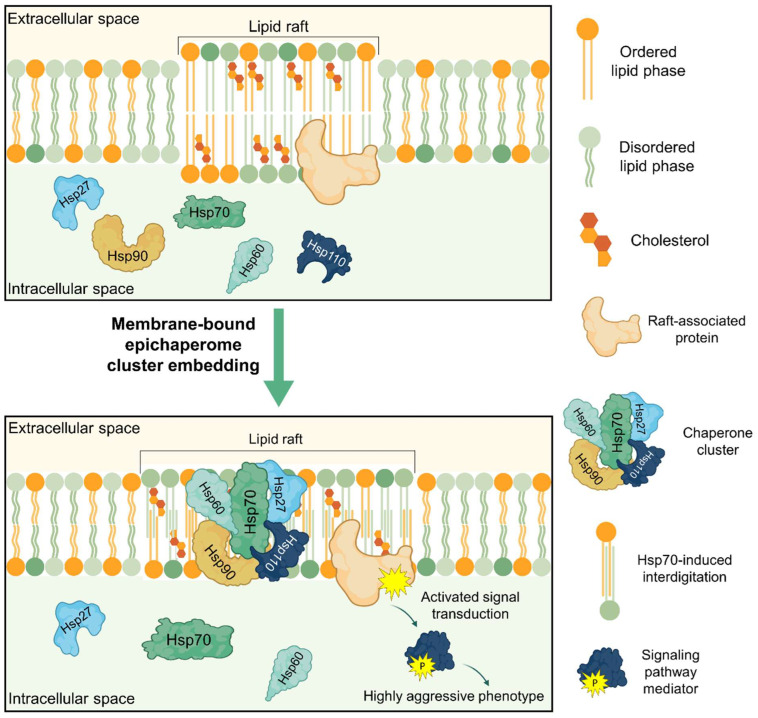
Potential role of membrane-bound epichaperome in cell functioning via alteration of membrane biophysical properties. By incorporating into ordered membrane domains of cancer cells, various members of HSPs can form a stable chaperone cluster and influence the biophysical properties of the lipid bilayer. For instance, HSP70 increases the area of ordered domains and induces interdigitation. HSP27, HSP60, and HSP90 reduce fluidity and increase rigidity. This leads to altered functioning of raft-associated proteins and activation of corresponding signaling cascades that promote the aggressive phenotype of cancer cells.

In vitro and select cellular studies indicate that chaperones can locally enhance lipid order. However, generalizing this to a dominant, cell-wide epichaperome regulator of membrane integrity is currently speculative and requires extensive in vivo and intact cell models. We propose that by modulating membrane physical properties, the chaperone complex stabilizes lipid rafts and supports enhanced signaling from embedded proteins, allowing cells to realize a highly aggressive phenotype (Figure 2). This intricate chaperone–lipid–effector protein interplay expands interactome networks, broadening chaperone functionality in cells. For cancer cells, the enhanced signaling and stabilized membrane platforms resulting in the chaperone–lipid effector protein reciprocal interaction may therefore serve as an additional protective mechanism, bolstering their survival and facilitating their dissemination. These findings demonstrate the inextricable linkage between the epichaperome and membrane biophysics and their mutual regulation. A comprehensive study of lipid-protein interactions will deepen our understanding of cellular events.

## 3. Plasma Membrane Lipid Heterogeneity and Chaperone Interactions

The heterogeneity of lipids in the plasma membrane shows a sterol-sphingolipid interaction that is driven by thermodynamically favorable chemical and geometric properties [65]. This property of the plasma membrane generates microdomains with distinct biophysical properties. In effect, these interactions overcome demixing costs, establishing stable, liquid-ordered domains within the liquid-disordered membrane that enable synergistic membrane functionality [2,66]. These spatiotemporally organized microdomains facilitate localized membrane reactions, architectural modulations, and curvature changes with minimal bioenergetic cost to the overall plasma membrane.

As we discuss later, both lipids and the heat shock proteins (HSPs) modulate the presence or behavior of one another.

The spontaneity and self-organization are hypothesized to be a potentially evolving process that requires membrane-associating proteins [67]. Importantly, lipid rafts tailor membrane identities and docking sites that function as molecular hubs for proteome-wide membrane interactions and trafficking. It is within these molecular hubs that HSPs reside, a localization that has been supported by demonstrable evidence [32,37,56,59,60,68,69,70]. It should be emphasized here that the HSP70, HSP90, HSP40, and HSP60 families have all been reported to interact with the plasma membrane. An interaction that is modulated by non-covalent interactions (electrostatic and hydrophobic) between HSPs and the lipid raft microdomains.

**Figure 3:**
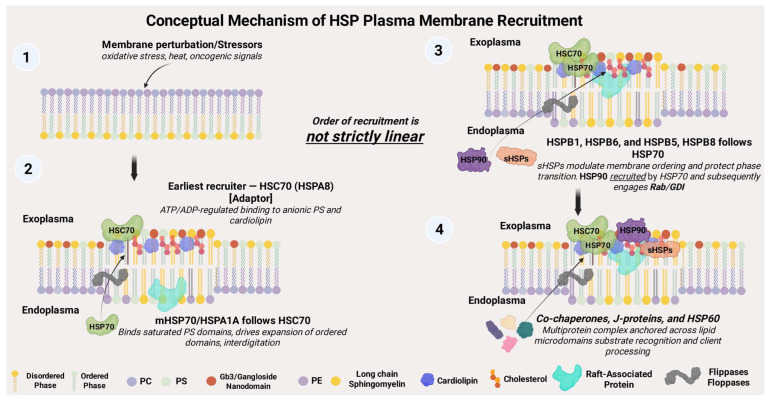
Conceptual Model of HSP Plasma Membrane Recruitment. Cellular stressors, including oxidative damage, heat, and oncogenic signals—trigger membrane perturbation, initiating a hierarchical yet non-linear recruitment of heat shock proteins (HSPs). HSC70 binds first, acting as an adaptor and ATP/ADP sensor. This facilitates membrane association of mHSP70/HSPA1A via phosphatidylserine and cardiolipin. Small HSPs (HSPB1, HSPB6, HSPB8) are subsequently recruited, forming multiprotein complexes with J family proteins (HSP40 family) and co-chaperones that stabilize membrane integrity and promote aberrant cell behaviors as exemplified in cancer.

The nature of the HSP–lipid raft interactions shows a high predilection for the highly negatively charged phospholipids, particularly phosphatidylserine (PS), palmitoyl-oleoyl phosphatidylglycerol (POPG), glycosphingolipid Gb3, sulfogalactosyl ceramide, and bis(monoacylglycero)phosphate (BMP) [71], all of which carry high negative charges. While not entirely positively charged, HSPs carry patches of positively charged amino acids (AA), hydrophobic regions, and polar AA that mediate hydrophobic and steer electrostatic interactions that are supported by lipid clustering [37,53,61,72]. Figure 3 shows a conceptual model of the order of HSP recruitment to the plasma membrane in response to stressors reported in the context of cancer.

As discussed later, regarding the role of HSPs in membrane dynamics during cellular transformation, a key focus concerns the functional significance of HSPs within the lipid raft microdomains. Understanding their role in these PM structures is essential for a comprehensive view of their impact on cellular adaptations. While raft-resident proteins, particularly receptors that mediate diverse cellular functions, rely on the lipid raft platform—a system partly modulated by the HSPs—we hypothesize that HSPs stabilize and control the aberrant interactome at the intra-extracellular signaling gateway via a dual mechanism: modulating the lipid raft composition and stability and the proteome-wide interactome in the raft. HSPs, potentially through chaperoning protein folding and preventing aggregation, maintain proteostasis. Furthermore, HSPs support the stability of the lipid raft platform, which is crucial for robust signal transduction. Thus, investigating HSPs within the heterogenous plasma membrane compartments offers new avenues for understanding and targeting cellular transformation, suggesting therapeutic strategies based on modulating HSP-mediated protein interactions.

## 4. Membrane-Bound Heat Shock Proteins

The heat shock protein family exhibits diversity at the cell surface, with various isoforms displaying unique characteristics and functions. While these membrane-bound activities are essential to maintaining normal cellular function, they can be hijacked in pathological cascades. Hence, understanding the specific HSPs at the cell surface is therefore paramount. This section provides an overview of identified membrane-bound HSPs in humans. To delve into the intricacies of their mechanistic activity at the PM, we refer you to our previous work [73] and related literature.

To date, membrane-bound HSPs have been observed across most HSP families, including sHSPs, HSP40, HSP60, HSP70, HSP90, and HSP100. HSP70 is particularly prominent, with diverse members displayed at the PM. The HSP70 family includes key isoforms such as HSPA1A (also known as HSP70), HSPA5 (also known as BiP or GRP78), HSPA8 (Hsc70), HSPA9 (GRP75), and HSPA1B, which is highly similar to HSPA1A [63,74,75]. These membrane-localized HSP70 family members support various membrane receptors, modulate diverse signaling pathways [76,77,78], mediate viral endocytosis [78,79,80], foster cancer stemness, invasion, and metastasis [63,81], and regulate procoagulant activity of tissue factor [82], highlighting their profound impact on membrane-associated signaling.

The HSP90 family, with HSP90 and GRP94/GP96 as prominent members [83,84], acts as a cornerstone for the cytosolic epichaperome network [63,85]. This pivotal role raises the question of their potential to nucleate and regulate a corresponding novel membrane-bound system. Beyond this structural significance, HSP90 members are recognized for their immunomodulatory function [86,87] and involvement in cancer progression [88,89].

Moreover, small HSPs, notably HSP22/HSPB8, HSPB1/HSP27/HSP25, and HSPB5/α-crystallin, have all demonstrated membrane-stabilizing effects [50,90,91], suggesting their contribution to substrate binding and overall stability within a membrane epichaperome complex. Their involvement presents another interesting area within this evolving field.

Finally, membrane-bound HSP40/60 has also been reported, further expanding the repertoire of HSPs involved in membrane adaptations [92,93]. Our recent discovery of an “mHSP cluster” composed of mHSP70, mHSP90, mHsc70, and mHSP105 [63] further underscores the involvement of the HSP110 family. Also, considering the HSP60/HSP10 functional dependence, HSP10 membrane colocalization could be plausible. The cumulative activity of these membrane-bound HSPs highlights their crucial role in proteome-wide dynamics, impacting cellular stress resilience.

While HSP integration into the cell membrane offers a beneficial counteractive measure to stress, differing effects of this incorporation are imminent, warranting more rigorous experiments that potentially reveal a complex, membrane-bound epichaperome. Elucidating the connectivity between this putative membrane-bound network and the established cytosolic and organelle-specific HSP systems is crucial for a holistic understanding of cellular stress response and therapeutic strategies. The imports of these studies will lead us to examine critical roles of HSPs in membrane fluidity, PM protein stability, and folding within this dynamic environment.

## 5. HSPs and Membrane Fluidity, Membrane Protein Stability, and Folding

The direct link between lipid composition and plasma membrane fluidity is unequivocally established. For instance, cis-saturated acyl groups tend to favor the formation of ordered domains, whereas trans-unsaturated acyl groups promote disorder and a more fluid membrane [94,95]. Equally, cholesterol modulates membrane fluidity based on a complex and concentration-dependent fashion: high exoplasmic leaflet cholesterol levels reduce fluidity by increasing lipid packing and decreasing lateral diffusion; depletion of cytoplasmic leaflet cholesterol rather increases fluidity [96,97,98]. Likewise, fluctuating external signals (temperature, osmotic pressure) induce configurational alterations (fluidization and rigidifying) that modulate specific microdomain ordering and influence overall membrane fluidity [99,100]. Moreover, receptor-specific ECM component interactions can also trigger lipidome remodeling, altering the lipid species distribution that translates into downstream signaling cascades [101,102].

The dynamic architecture of the PM also shapes protein folding and stability. The effect is driven by lateral pressure, membrane curvature, and lipid deformations. These factors are reinforced by the presence of unsaturated lipid and cholesterol [103,104].

Contrary to the membrane fluidity, lateral heterogeneity imposed by sterol and sphingolipid scaffold proteins promotes signaling, while anionic phospholipids (PS, PI) modulate protein structure and function through electrostatic forces, influencing membrane protein partitioning. A precise zwitterionic/anionic lipid balance maintains membrane architecture, affecting curvature and stability [105]. Superimposed on this intricate model, HSPs modulate membrane properties through chaperone activity and specific lipid domain interactions.

Illustratively, in a liposome-based assay, Hsc70/HSPA8 bonded with striking specificity to negatively charged PS and cardiolipin-rich microdomains—rich in unsaturated phospholipids—while binding with low affinity for phosphatidylcholine. Binding was ATP/ADP-dependent, promoting HSPA8 oligomerization via disulfide bridges at the membrane interface [42]. Subsequently, HSP90AA1 coincubation with PS/cardiolipin liposomes in the presence or absence of HSPA8 and analysis by LDS-PAGE revealed HSP90AA11 retained on the liposomes only in the presence of HSPA8. These observations possibly indicate that HSPA8 drags HSP90AA1 into the unsaturated phospholipid regions of the plasma membrane [42]. But the direct recruitment of HSP90 by HSPA8 in intact plasma membranes remains to be validated. HSP22 binds the inner leaflet phosphatic acid, PI, and PS of the small unilamellar vesicle model system [57]. HSPB1/HSPB5 also interacts differentially with different membrane domains [52], while liposome assays demonstrate HSPB6 lipid-dependent chaperone activity in PS/cholesterol-rich sites [58]. In a thylakoid liposome membrane model, HSP17 and α-crystallin interact and stabilize monogalactosyldiacylglycerol and phosphatidylglycerol isolated from *Synechocystis* thylakoids, extending the temperature range of its fluid state [90]. The sHSPs Lo18 from *Oenococcus oeni* and HSP1 and HSP3 from *Lactobacillus plantarum* have also been shown to control the membrane fluidity and physicochemical properties (membrane packing) by modulating membrane stability in synthetic bilayers [106]. These interactions induce conformational changes in membrane structure and fluidity, fostering cellular function and stress acclimation.

Remarkably, the potential role of HSPs in cancer development has become an area of intense interest. It is hypothesized that the increased insertion of HSPs into the lipid membrane is a critical step in the biogenesis and development of an aggressive cancer cell phenotype [40,54,63,73]. This is particularly relevant when considering the formation of membrane-bound epichaperome with a range of malignant properties [107].

To sum up, the PM is a living mosaic—its lipids and proteins are continuously tuned by HSPs to meet the cell’s changing needs. Membrane fluidity, a critical biophysical parameter, frames protein function and stability, enabling dynamic interactions and signal transduction. Simultaneously, HSPs actively remodel the membrane landscape, adapting it to cellular stress, modulating protein conformers, and, in some instances, influencing disease progression. Targeting HSP–membrane interactions thus offers novel therapeutic avenues, from oncological interventions to neurological and reproductive health.

Innovative strategies could precisely manipulate HSP–membrane interactions by selectively targeting distinct lipid microdomains or allosterically modulating HSP ATPase activity at the membrane interface, as suggested by László Vigh and others. Beyond this, approaches such as engineering tailored nanodomains with specific sterol and sphingolipid compositions, employing peptidomimetic inhibitors that disrupt HSP–lipid binding, or selectively inducing the release of aberrant membrane-bound HSP-containing vesicles offer further therapeutic opportunities. Finally, advanced delivery systems utilizing stimuli-responsive liposomes or exosome-based carriers promise precise and spatially confined modulation of these critical molecular interactions, ultimately paving the way for targeted membrane lipid therapies.

## 6. Chaperone–Lipid Membrane Trafficking

### 6.1. HSPs and Ion Channels

The plasma membrane, as a crucial barrier, relies on ion channels to control ion flux [108]. These channels function as regulated gateways, modulating a myriad of cellular processes. While the established role of ion channels as pore-forming proteins is undisputed, the involvement of HSPs in ion channel function has been a subject of significant and evolving investigation. In this section we explore the relationship between HSPs and ion channels, ranging from direct channel formation to chaperone-mediated regulation of channel activity and trafficking, while also highlighting the complex bidirectional regulation between cellular stress and neuronal excitability.

Reconstituted bilayer and liposome experiments report HSC70/HSP70-dependent conductance in vitro. The physiological existence of HSP-formed channels in native membranes remains unproven and requires native single-channel recordings, acute depletion/rescue, and non-conducting mutant controls.

HSPs can directly insert into the lipid bilayer and form ion channel-like pores. In all these studies, reconstituted membrane (planar lipid bilayers and liposomes) model systems have been employed. As proposed by Arispe and De Maio, Hsc70 directly integrates into the PM, forming ATP/ADP-dependent cationic channels [109]. A similar report by Vega et al. demonstrated the same phenomenon in macrophages [60]. It was also demonstrated that HSP70 oligomerization and clustering at the PM increases cellular uptake [61]. At the same time, we have also shown that HSP70 oligomerization upon PM interaction induces channel formation that modulates ion permeability and plasma membrane (PM) electrical stability [54]. The readouts from these studies have shown characteristic single-channel currents (patch/voltage clamp), dye leakage, and conductance changes. These findings expand HSP functionality and support direct lipid interaction mechanisms, suggesting an intrinsic and structural regulation capacity beyond chaperoning.

HSPs also directly tune ion channel activity. The role of the HSPs here includes the modulation of ion channel protein maturation, stability, and plasma membrane delivery of endogenous ion channels. The approach in most of these studies utilizes cellular and animal-based models. For instance, HSP70 can bind directly and activate K^+^ channels in a Ca^2+^-dependent manner, shifting current-voltage along a negative potential [110]. This affects gating, modulating K^+^ influx and membrane potential. Moreover, in response to neuronal signal transmission, Hsc/HSP70 directly interacts with synaptic vesicular monoamine transporter-2 (VMAT2) [111], which collaboratively interacts with tyrosine hydroxylase and aromatic amino acid decarboxylase to regulate vesicular loading, storage, and the subsequent release of monoamines [112]. As one would expect, denatured Hsc/HSP70 exerts no effect on VMAT2. Similarly, Hsc70 and a distinct class of J domain proteins, the CSPs, anchor to GABAergic synaptic vesicles [113], possibly mediated by L-glutamic acid decarboxylase (GAD) [114]. This complex facilitates the functional packaging of GABA via VGAT, with VGAT and Ca^2+^/calmodulin-dependent kinase II forming a complex with GAD for GABA release. Collectively, HSC70/HSP70 and their co-chaperones are key for neuronal action potential regulation.

Furthermore, CSPs, as part of the activities, play integral roles in the regulation of the molecular mechanisms underpinning P/Q and N-type Ca^2+^ channel exocytosis [115,116], coupled with HSP/Hsc70 ATPase activity [117]. Also, Ca_V_1.2 Ca^2+^ channel cell surface trafficking relies on the collaborative activity between kinesin and HSP70/90 [118].

In addition to direct interactions with ion channels, HSPs influence ion channel quality control, ensuring proper folding, assembly, trafficking, and overall functional integrity. Canonical ion channels have precise structures, which is at odds with the known, flexible conformations of HSPs. Thus, the concept of direct HSP-mediated channel formation demands a rigorous and definitive validation.

Moreover, the human ether-a-go-go-related gene (HERG) K^+^ channel, vital for cardiac repolarization [119], exemplifies this. Importantly, HERG channel maturation, stability, and precise trafficking rely on a vast network of HSP/Hsc70, HSP90α (which also prevents CHIP binding to ensure HERG maturation [120]), 14-3-3, cyclophilin A, Hdj-2, Hop, Hip, Bag-2, FKBP8, and calnexin [121]. This network is tightly regulated by HSF1 and is indispensable for QT wave potential [122].

Again, the CFTR channel, essential for epithelial water and ion balance [123], presents another illustrative example. While primarily synthesized in the ER and trafficked to the PM [124], although other non-conventional ER-PM pathways exist, their PM translocation is facilitated by Hsc70, HSP40 family analogues (human DnaJ1/2 (Hdj2)) [125], ERp29 [126], FKBP38 [127], CHIP [128], BAG-1 [129], HSP90, and Aha-1 [130,131]. Strikingly, Hsc70, DNAJB2, Aha-1, or ERp29 ablation paradoxically increases CFTR surface density, while DNAJA1, BAG-1, or HOP knockdown decreases it. Additionally, the role of HSPs extends to systemic responses where Ca^2+^ channels actively respond to heat stress by activating the HSR, thereby regulating HSP levels in a fascinating feedback loop [118].

The readouts of the HSP effect on folding/trafficking of native ion channels have generally been characterized by pulse chase maturation, co-immunoprecipitation, and functional patch clamp in intact cells. Strikingly, the pharmacological inhibition of HSPs typically reduces the maturation of channel abundance and their surface residency.

While the concept of HSP forming de novo ion channels remains a frontier, it requires solid experimental validation. However, their profound and established chaperone role is irrefutable in the activities of ion channel activity. They are emerging as central, multifaceted regulators of cellular excitability, exerting their influence through a sophisticated repertoire that spans from chaperone-assisted maturation to direct, functional modulation of ion channels.

### 6.2. Transcytosis

Transcytosis acts as a cellular bypass, selectively transporting essential cargo across the otherwise impermeable PM barrier, often entering through the apical side and exiting through the basal membrane on the other end of the cell. This precisely regulated process ensures that molecules, from nutrients to signaling factors, are delivered to their intended destinations, enabling targeted communication and resource allocation. Here, we examine the few conducted works that directly detail HSP-transcytosis.

The transepithelial translocation of *Listeria monocytogenes* offers a compelling illustration, as *L. monocytogenes* infection-associated stress co-opts the upregulation of membrane-bounded and intracellular HSP60 expression in enterocytes [132]. This strategy relies on *L. monocytogenes* secretome: Listeria-associated protein (LAP), a critical virulence factor that directly binds and activates its membrane-bounded HSP60 receptor [132,133,134], promoting *L. monocytogenes* translocation across the epithelial barrier [135]. Effectively turning a physiological pathway into a gateway for establishing systemic infections. Here, the role of HSPs in the transcytosis process is clearly facilitative, leading distinctly to a pathological outcome.

However, HSPs can also act to restrict transcytosis. Targeting the rhomboid intramembrane protease (iRhom1) is a promising approach to enhancing chemo-immunomodulatory therapies [136,137]. In a report by the Song Li group [137], HSP27 has been implicated in regulating vesicular formation and translocation of CD44-targeting nanocarriers across the blood–brain barrier by targeting iRhom1. By regulating cellular transcytosis, HSP27 preserves the functional integrity of tight junctions, maintaining junctional electrical resistance [138]. This demonstrates that HSPs, like HSP27, can be exploited in the transcytosis pathways to corroborate therapeutic efforts. Nonetheless, further mechanistic dissection is needed to confirm direct targeting.

These contrasting examples show that while HSP60 is co-opted by pathogens to promote translocation across cellular barriers, HSP27 can act to maintain barrier integrity by regulating vesicular trafficking and tight junction functions.

### 6.3. Endocytosis

Endocytosis, a fundamental and highly conserved cellular mechanism, remains crucial for cellular adaptation through biomolecular internalization, membrane remodeling, and stimulus response. Initiating with primary endocytic vesicles (PEVs), early endosomes (EE) fuse and traffic endocytic cargoes—pinocytic and phagocytic vesicles—to distinct intracellular locations. Acting as a pivotal modulator of endosome dynamics, the endosomal sorting complex required for transport (ESCRT) directs the biogenesis of the late endosome maturation and controls the endosome invagination (ILV) (particularly important as the source of exosome formation upon their membrane secretion). Subsequently, Ees follow one of three paths: recycling to the PM; maturation and fusion with endolysosomal fusion; or transformation into multivesicular bodies (MVBs) containing intraluminal vesicles (ILVs), which can be released extracellularly as exosomes.

While endocytosis is clearly essential for endosome and exosome biogenesis, the precise mechanism that determines the selective sorting and targeting of cargo into these vesicles represents an intense area of ongoing research. Among the factors modulating endocytosis, HSPs play a crucial role. Cell studies report correlations between mHSP70 cluster size and increased endocytotic uptake in cancer cells [61]. These observations support an association but do not alone establish direct causality without acute manipulation and rescue experiments.

HSPs, mostly HSC70 and HSP90, facilitate endocytosis through mechanisms including cargo recognition, selection/sorting, and cargo packaging and secretion, collectively culminating in a range of cellular processes, including immunological surveillance, nutrient uptake, receptor internalization and signal modulation, and the control of cell adhesion and migration. The following sections will delve into the specific roles of HSPs in endosome and exosome dynamics.

### 6.4. HSPs and Endosome Cargo Selection

The endosomal recycling pathway, a critical component of cellular homeostasis and signal transduction, requires the influence of HSPs. HSPs, specifically the ubiquitously expressed Hsc70 and HSP90, characterized by their high basal expression, are indispensable across the endocytic continuum. Their multifaceted roles range from initiating receptor-mediated endocytosis and facilitating clathrin coat assembly to driving late endosome maturation and protein sorting. Of note, HSP70, as a functionally overlapping homolog to Hsc70, can also effectively mediate these essential chaperone functions within the endocytic pathway. These chaperones with their inherent capacity to critically chaperone cellular emergence underscore their roles.

In immune activation and surveillance, the selection of endosome cargo influences key signaling pathways. In the process, HSP90 plays a critical role, influencing the endosomal cargo selection, particularly in plasmacytoid dendritic cells (pDC) through TLR9 activation triggered IFN-α secretion via endocytosis [139]. A study by Okuya and colleagues highlights that HSP90 chaperones self-DNA and the CpG-oligodeoxynucleotides complex (HSP90/DNA/CpG-ODN complex) and directs them to Rab5^+^ early endosomal Ag 1^+^-static endosomes. This targeted delivery is indispensable for efficient TLR9 signaling and IFN-α secretion in early endosomes; it drives pDC maturation in late endosomes [140]. Importantly, HSP90 is crucial for retaining CpG-ODN within these static early endosomes and critical for converting human DNA into potent IFN-α secretion activators [140,141]. Consistent with this, HSP90-peptide complexes are strictly sorted and cross-presented via MHC class I molecules in Rab5^+^, early endosome effector protein (EEA1^+^) static early endosomes [142,143], emphasizing the involvement and importance of HSP90 in the regulation of these processes.

Related to the LE processing, which has the feature of cytoplasmic cargo fusion, drives the formation of MVBs through ILV events. LE cargo fusion is ESCRT I/III and Vps4 complex controlled [144,145]. While HSPs exert significant influence, the endosomal network also relies on complex interactions beyond direct HSP activity. Of note, Hsc70 interacts with LE phosphatidylserine-rich membrane domains via its substrate-binding domain lysine cluster to establish vital electrostatic contacts. Remarkably, this Hsc70/PS interaction mediates the targeted enrichment of LEs with KFERQ-containing proteins through intraluminal invaginations, effectively driving microautophagy [146,147].

The maturation from PEV to the late endosome involves the complex interplay of the Rab GTPases and the ESCT family proteins, which are critical for these stages. Thus, the dynamic localization of HSPs to the plasma membrane and along the maturation routes in physiological and cellular stress states suggests a broader, yet-to-be-fully elucidated role beyond their established functions. These HSP activities require a complex interconnection with co-chaperones and other chaperones. This coordinated effort may forge a dynamic HSP interactome along the endosomal maturation path all the way up to the formation and release of exosomes.

### 6.5. HSPs and Exosomes

Exosomes are nanoscale vesicles (~100 nm average) formed by multivesicular bodies (MVBs) fusing with the PM, releasing intraluminal vesicles (ILVs) into extracellular space. Enriched with lipids, cytosolic and membrane-bound proteins, DNA, mRNA, miRNA, and metabolites [148,149], exosomes are integral to maintaining cellular homeostasis [150], mediate cellular signaling, and serve as tools for programmed cellular communication [148]. With endosomes as their precursors, their biogenesis, cargo sorting, and secretion are tightly regulated by the ESCT complex, Rab GTPases, the SNARE complex, and the syntenin-ALIX-syndecan axis [151,152]. These regulators orchestrate ILV formation, cargo packaging, MVB maturation, and exosome release. HSPs like Hsc70, HSP90, and αβ-crystallin chaperone, acting as key constituents of the exosomal machinery [153,154], play critical roles in exosome biogenesis, cargo sorting, and secretion.

These chaperones modulate the endocytosis-MVB-exosome pathway at multiple interconnected levels. Their influence is both direct and associative. Directly at the plasma membrane, HSPs interact with the Rab GTPases to sustain vesicle trafficking and MVB maturation [155,156,157,158]. Concurrently, they cooperate with ALIX to direct selective protein incorporation [159]. Following MVB maturation, HSPs promote SNARE assembly for efficient MVB plasma membrane fusion, ensuring robust exosome release [160,161,162,163]. HSPs also regulate the rate of MVB formation and secretion [164] and play a significant role in stress-response regulation [165]. Associatively, their enrichment as exosome cargos and markers has been correlated with tumor progression, immune modulation, and biomarker potential, though these remain largely correlative observations [166,167,168,169,170].

Given the importance of Rab GTPase in the early exosome pathway, their periodic membrane recycling is crucial for continued function. Following their activation and GTP hydrolysis, Rab GTPase must be efficiently retrieved from the membrane for subsequent vesicle trafficking. Facilitating Rab recycling, HSP90 interacts with the guanine nucleotide dissociation inhibitor (GDI) complex [155,156,157]. This interaction is critical as GDI extracts inactive, GDP-bound Rab GTPases from membranes, maintaining them in a soluble, cytosolic state for subsequent reactivation. Also, the HSP90/GDI complex drives VSV-Gts trafficking, enhancing Rab1-mediated ER-to-Golgi and intra-Golgi transport, vital for Rab1 membrane retrieval and exosome pathway function. Thus, HSP90/GDI-mediated Rab GTPase recycling ensures sustained and efficient exosome formation with appropriate cargo.

Moreover, Géminard et al. also demonstrated that Hsc70 indirectly regulates exosome cargo sorting [159]. Their work showed that, in the absence of the AP2 adaptor complex, Hsc70 binds directly to TfnR, along with ALIX, to effectively control exosome cargo sorting [159]. Hence, AP2 adaptor complex degradation during reticulocyte maturation allows Hsc70/TfnR interactions to target specific proteins, like TfnR, for preferential exosomal release.

In neuronal vesicle secretion, SNARE proteins and VAMP regulate exosome fusion with the cytoplasmic surface for extracellular release [160,161]. Chaperoning the smooth transition of this activity is cysteine string protein α (CSPα) CSPα/Hsc70/SGT. Functionally, CSPα/Hsc70/SGT binds directly to monomeric SNAP-25, a SNARE protein. This CSPα/Hsc70/SGT/SNAP-25 complex prevents SNAP-25 aggregation, crucial for SNARE complex assembly [162,163]. In addition, the inhibition of the sHSP, αβ-crystallin, has been tied to exosome secretion inhibition. In effect, αβ-crystallin inhibition disrupts MVB plasma membrane trafficking but enhances endolysosomal activity, decreasing exosome secretion [164]. Moreover, in myocardial infarction, hematopoietic stem cells (Sca-1^+^ stem cells) exhibit peak HSP70 expression, which is dependent on miR-34 repression. Hsf1 epigenetically represses the miR-34a promoter, contrasting its direct upregulation of HSP70 expression, suggesting a stem cell-specific regulatory mechanism. This Hsf1-miR-34a/HSP70 complex fosters the release of exosomes from transplanted Sca-1+ stem cells, promoting ischemic cardiomyocyte survival, ultimately restoring global heart function [165].

Lastly, the identification of HSPs as exosomal markers and cargos [153,166,167,168,169], and the presence of HSP70 in tumor-derived exosomes underscore their potential as diagnostic and therapeutic targets, particularly in cancer, where exosomal HSP70 is emerging as a biomarker for tumor prognosis [170].

While HSPs demonstrably influence exosome formation and function, the complexity of exosome biogenesis necessitates acknowledging our limited understanding and potential for HSP-independent mechanisms. Exosomal HSP function, including contradictory immunostimulatory/suppressive effects, warrants caution in therapeutic exploration [171,172,173,174]. Preclinical studies suggest that HSP70 exosomes can promote tumor growth by inducing Stat3 signaling in MDSC [171,172]. However, the translational application of exosomal HSP70 as a biomarker is hampered by standardization and calibration challenges across populations. As such, future studies must diversify populations to account for wider exosomal HSP70 profiles. Finally, detailed mechanistic investigations are needed to clarify the contradictory effects of exosomal HSP70 to guide safe therapeutic strategies.

### 6.6. HSPs and Exosome Uptake and Transfer

Extracellular vesicles (EVs), including exosome release and uptake, initiate a cascade of emergent behaviors in recipient cells through the transfer of bioactive cargo [175]. The uptake process is guided by diverse targeting mechanisms, ranging from direct exosome-recipient cell receptor interactions and endocytosis to micropinocytosis and phagocytosis, with exosome size influencing the uptake efficiency [149,176]. Exosome release and uptake are hypothesized to be governed by a combination of stochastic and deterministic processes, where the releasing cell specifically targets a particular recipient. Yet, while exosome uptake hinges on membrane proteins and receptors, the precise mechanisms remain incompletely understood, suggesting the potential role of HSPs considering their indispensable roles in chaperoning most cellular processes.

Indeed, accruing evidence points to significant contributions from HSPs in the exosome uptake process. Svensson et al. demonstrated that exosomes can interact with and phosphorylate ERK1/2 and HSP27, key signaling molecules within the lipid raft and sorting platforms [62]. These interactions underpin the reliance of exosome uptake on intact ERK1/2-HSP27 signaling, which is further complicated by the observation that caveolin 1 negatively regulates ERK1/2 phosphorylation during exosome internalization by recipient cells, suggesting a connection between these pathways and the lipid rafts. The need for HSPs in exosomes extends beyond mammalian systems, as illustrated in *Saccharomyces cerevisiae*. Here we have come to understand that exosomes containing the HSP70 ortholog stress-seventy subunit A2 (Ssa2) transfer thermotolerance to neighboring cells during heat stress. However, mutant cells lacking Ssa2 produce exosomes that cannot confer thermotolerance, highlighting Ssa2’s essential role in maintaining the exosome characteristics and enabling the transfer of vital bioactive cargos and traits [177].

Finally, quantitative characterization of EV uptake and content delivery within mammalian cells has demonstrated the central role of HSP90 [178]. HSP90 inhibition reduces the rate and quantity of EV internalization, specifically via lipid raft-mediated endocytosis. Interestingly, hindering HSP90 activity also impedes EV cargo release. This positions HSP90 as a key regulator of intercellular communication, modulating membrane remodeling, vesicle trafficking, and fusion.

Notwithstanding these advances, the exact mechanistic details of HSPs in the workings of lipid raft remodeling in response to exosome uptake remain a compelling area for further research. We propose that further studies should look at lipid/protein changes occurring within the PM after exosome uptake, assessing the impact of mHSP90/mHSP70/sHSPs in these changes. Moreover, investigations into how manipulation of lipid raft constituents affects HSP90’s role in the uptake process would be very enlightening. By addressing these questions, our understanding of exosome-mediated intercellular communication could enhance the engineering of exosomes for a range of diseases.

### 6.7. HSPs and Tunneling Nanotubes

Tunneling nanotubes (TNTs), nano-sized membranous conduits (50–200 nm), mediate intercellular interactions [179]. Interestingly, some cells can extend TNTs over distances exceeding several cell diameters, creating structural cellular networks [180]. This cellular structure allows for the rapid and targeted exchange of information, bypassing canonical signaling systems and pathways. TNT formation and function involve HSPs and membrane-associated proteins like RalA GTPase [181], Rho GTPase [182], Rac-1/Exocyst complex [183], and Msec/TNFαIP2 [184,185]. This intricate process facilitates the transfer of cellular components, including nucleic material [186,187], proteins [188,189], neurotransmitters [179], toxic elements [190], and even entire organelles [191,192].

As demonstrated in our recent paper [64], mHSP70 plays a vital role in TNT formation and activity within lipid raft microdomains, specifically globotriaosylceramide (Gb3)-rich lipid rafts as depicted by STED microscopy. Expectedly, mHSP70 inhibition with methyl-β-cyclodextrin and lipid raft microdomain pulldown causes cytoplasmic translocation of mHSP70 and the resultant depletion of TNT structure. Deductively, the dynamics of mHSP70, as we demonstrated, presuppose a link between mHSP70 and TNT formation in the lipid raft microdomain. But the upstream regulatory mechanisms and causal links involved here remain speculative.

Moreover, the tumor necrotic factor-α-induced protein 2 (TNFAIP2/MSec) (N-termini binds) interactions with the RalA/exocyst complex modulate membrane deformations vital for TNT formation [184,193]. Intriguingly, the conformational stability and catalytic activity of TNFAIP2, involving phosphoinositide binding at its N-terminus and RalA activation at its C-terminus, are ERp29 and connexin-43 dependent. As proof of concept, ERp29 inhibition reduces TNFAIP2 protein levels and its RalA/exocyst complex formation, making TNFAIP2 ERp29’s key target [193].

Furthermore, HSPs, glutathione, and membrane proteins, including RalA GTPase, coordinate remarkable responses to reduce copper stress [190]. Together, they modulate the copper transporter, ATP7A, which drives the intracellular and intercellular efflux of copper in oyster hemocytes via TNTs to reduce copper overload. This highlights potentially therapeutic targets. As such, researching HSP’s mechanisms in copper metabolism disorders could unlock insightful translational applications.

The connection between TNTs, mHSP70, TNFAIP2, ATP7A, and ERp29 points at a picture of cellular communication and stress response. However, the upstream regulators of TNT formation, cargo loading, and the mechanistic roles of membrane-bound HSP remain enigmatic. It also remains to be explored if TNT manipulation could offer a therapeutic potential. Moreover, TNT dependence on GB3-rich lipid rafts and phosphoinositide-interacting proteins links to biogenesis of lipid metabolism. Raising the key question, how do changes in lipid synthesis and membrane composition impact HSP and TNT regulation? Could specific lipids trigger or inhibit TNT formation? Exploring these questions could provide informative insights into the biology of TNTs.

## 7. Lipid Synthesis and HSP Dynamics

The regulation of lipid synthesis involves a complex reciprocity of factors, with sterol response element binding proteins (SREBPs) acting as central transcriptional regulators. HSPs dynamically control the process by interacting with the master regulator, SREBP, which directly interacts with lipid-metabolizing enzymes, adapting the process in response to environmental stressors, and through positive and negative feedback responses.

As the master regulator, SREBP regulates lipid synthesis by controlling the expression of lipid-metabolizing enzymes [194,195]. Under cholesterol depletion, SREBPs complex with SREBP cleavage activation protein (SCAP) (SREBP/SCAP complex) and the ER/lipid droplet-associated protein Cideb, which promotes the loading of the SREBP/SCAP complex into COPII vesicles essential for its ER-Golgi escort [196]. Insulin-induced gene (INSIG) proteins sequester SCAP, preventing SREBP/SCAP complex transport to the Golgi and proteolytic processing [197]. Here, HSP90 stabilizes the SREBP/SCAP complex through their C-termini, potentiating SREBP-mediated transcriptional activation of key lipogenic genes such as *FASN*, *ACC1*, *LDLR*, and *SCD1*, thereby controlling lipid synthesis [198,199]. However, pharmacological inhibition of HSP90 with 17-AAG increases the expression of the fatty acid and cholesterol biogenesis genes, including HGGCS1, *FASN*, and HMGCR [198], necessitating investigation into the specific axis in which HSP90 mediates lipid biogenesis.

Furthermore, high glucose stress upregulates Hsc70/HSPA8, which in turn restrains SREBP activity by blocking SCAP-mediated ER-Golgi transport [200]. HSPA8 modulates SREBP expression by facilitating its degradation via the CHIP-mediated ubiquitin-proteasomal pathway involving protein kinase R (PKR), thereby linking chaperone activity to lipid biosynthetic control. As a downstream effector of HSPA8 in SREBP regulation, PKR modulates INSIG1/INSIG2 phosphorylation, controlling the INSIG/SCAP interaction necessary for SCREB/SCAP ER-Golgi translocation [200].

Our understanding of lipid synthesis regulation reveals an interconnected relationship between SREBPs and HSP, showing a bidirectional interactome and regulatory circuit as opposed to a unidirectional pathway. While HSP90 and HSPA8 influence SREBP activity, SREBPs also control the expression of HSPs, highlighting a reciprocal regulatory loop in lipid homeostasis.

In response to heat shock or fluctuating sterol levels, SREBPs regulate DnaJA4 mRNA levels, showing a coordinated expression pattern with SREBP target genes, including *LDLR* and *HMGCR*. Conversely, when COS cells were genetically modified with a dominant-negative SREBP2 construct (delivered by an adenovirus construct), the observed changes in DnaJA4 expression were abolished. This outcome indicates that SREBP directly controls DnajA4 gene expression [201].

Additionally, more direct HSP–lipid enzyme interaction activity and levels have been reported. In a preclinical non-alcoholic fatty liver disease animal model study, Zhang et al. highlighted a correlation between fatty acid synthesis enzymes and HSP70 activity [202]. HSP70 expression was upregulated in fatty liver, promoting lipogenesis and lipid accumulation by increasing *SREB1C* and *ACC* mRNA levels, while HSP70 knockdown significantly downregulated fatty acid synthase (*FAS*), *ACC*, and *SCD* mRNA levels [202].

A recent study led by Chengkai Dai presented new evidence that HSF1 [203] and not HSP90 regulates *AMPK* and *Acc* [204] to regulate fatty acid metabolism. Knowing that AMPKα/γ are client proteins of HSP90 adds another layer of complexity to the dynamics of HSP regulation of lipid synthesis and homeostasis. We posit that the dynamic and transient nature of protein–protein interactions and the approach of cell lysis employed in ascertaining proximity interactions require reconsideration for approaches that allow for the preservation of the spatiotemporal pattern of analytes, as suggested in our earlier publication [107].

Summing up, the regulatory landscape of SREBP and lipid synthesis enzymes and the HSP axis are a dynamically regulated, bidirectional process that perpetually responds to the cellular environment [205]. External stimuli, such as heat shock, significantly reshape the intracellular lipid landscape, leading to increases in sphingolipids, fatty acids, and glycerophospholipids, while sterols stay relatively stable [205]. Cellular mechanics, such as ECM stiffening and RhoA-mediated actin-myosin contraction, are likewise crucial modulators of SREBP activity via AMPK activation [199].

Over and above, further studies looking into HSPs-lipid synthesis mechanisms would be enlightening. Key questions remain regarding HSP isoform coordination across different cellular compartments. Clarification of how HSPs sense and respond to lipid environment shifts and understanding how mechanical cues and external stimuli integrate with HSP-SREBP signaling are equally essential. Equally important is the development of therapeutic strategies to modulate HSP activity to restore lipid homeostasis in diseases such as NAFLD. Insights into these would illuminate HSP’s role in PM metabolism and modification.

## 8. HSPs and Plasma Membrane Lipid Remodeling

Plasma membrane biogenesis is orchestrated without dedicated lipid synthesis genes. Instead, a programmed system ensures the integrity of its key constituents through metabolic control. Importantly, the lipid membranes are actively and constantly remodeled, rather than synthesized de novo. As proposed by the Keller group [206], primordial plasmalemma co-localizes with amino acids through a positive feedback loop that plays a significant role, including membrane maintenance and remodeling. Within this context, HSPs have been proposed and supported by literature as key regulators of the plasmalemma metabolism and modification process. In this section, we now explore how HSPs actively modulate the PM lipid metabolism and drive essential modifications for cell survival.

HSP70, through multiple mechanisms involving direct interactions with PM constituents and enzymes [35,207,208], critically regulates PM dynamics and metabolism. Yet, the specificity and functional consequences of HSP70 PM regulation are cellular context- and HSP70 isoform-dependent. For instance, the HSP70 can bind to specific membrane components, promoting its catalytic activity [35,207,208]. In isolated *Synechocystis* spp. thylakoid preparations, Thurotte et al. report that DnaK3 is essential for thylakoid membrane biogenesis and photosynthesis ratio maintenance under light/dark cycles [209]. Given the established light-dependent nature of thylakoid membrane development [210], DnaK3-deficient strains exhibited a significantly altered photosystem I-to-II ratio, reduced thylakoid layers and chlorophyll content, and enhanced adaptation in low-light conditions [209]. In another informative instance, HSP70-3’s interaction with PM-localized phospholipase Dδ (PLDδ) in *Arabidopsis thaliana* is crucial for thermotolerance. Detailing the mechanism, HSP70-3 translocates to the PM, inhibits PLDδ activity, fine-tunes phospholipid metabolism, and triggers cortical microtubule reorganization that safeguards cellular homeostasis under heat stress [211]. Conversely, inhibiting HSP70 membrane activity disrupts lipid metabolism by inhibiting acid sphingomyelinase activity, leading to lysosomal membrane destabilization and cell fate control, potentially activating cell death cascades [212].

In effect, the overarching function of HSP70 and its paralogues modulates membrane phospholipids, promoting emergent cellular behavior. Our recent findings demonstrate that HSP70/HSP70A1A remodels phosphatidylserine-rich domains, impacting enzyme activity, signaling protein recruitment, and membrane trafficking to control localized metabolic processes [54].

Apart from HSP70, the sHSP represents another class of molecular chaperones with evident plasma membrane activity, significantly influencing the membrane properties and lipid metabolism [52]. Csoboz et al. demonstrates that the sHSP, HSPB1, regulates lipid packing in cell membranes, maintaining high membrane order in the phase of membrane perturbations [52]. HSP27/HSPB1 interacts specifically with ceramide synthase 1 (CS1) and inhibits its activity while regulating mitochondrial membrane fluidity and stability [213,214]. Biologically, this prevents ceramide accumulation in the mitochondrial membrane, but HSP27 functional inhibition disrupts sphingolipid-mediated cellular activity and induces mitophagy in response to excessive ceramide accumulation [213,215].

Together, these findings paint a picture of HSPs as active players in lipid metabolism—not just passive responders to stress. HSP70 regulates membrane adaptation, while sHSPs, such as HSPB1, fine-tune lipid packing. This tight coupling suggests a convergence in intracellular lipid organization, potentially involving lipid droplets, and the formation of a protective epichaperome complex in pathological states. The diverse roles of HSPs in regulating PM lipid metabolism, from broad adaptations to fine-tuning lipid packing, underscore their crucial contribution to cellular homeostasis and resilience.

## 9. Lipid Degradation, Lipid Droplets, and Epichaperome

Lipid droplets (LD), morphologically dynamic organelles derived from the ER, maintain cellular lipid homeostasis by buffering potentially toxic lipid species [216]. These organelles consist of a neutral lipid core, primarily triacylglycerols and steryl esters [217,218], enveloped in a tightly regulated single phospholipid monolayer, exhibiting a continuum of size (0.1 µm–100 µm) and compositional plasticity across different cell types and metabolic states. Critical to LD biogenesis and maturation are Seipin and Promethin/Lipid droplet assembly factor 1 (LDAF1), which mediate inter-organelle interactions (<30 nm proximity) and lipid exchange at ER-LD membrane junctions through shuttle and bridge proteins without direct fusion [219,220,221,222,223]. Critically, these mechanisms show LDs as intricate platforms for lipid metabolism and integral energy regulators in response to cellular needs.

In the complexities of the LD, the HSPs have emerged as active regulators that influence LD fate, impacting LD biogenesis, degradation, and lipid transport. For example, HSP70 stabilizes FSP27, counteracting AMPK-mediated destabilization and degradation, thereby promoting the formation of large LDs for efficient lipid storage [224]. And as reported by Robichaud et al., HSP90AA1, HSPB1 (HSP27), HSPA5 (HSP70), and HSPH1 (Grp78) modulate cholesterol efflux from macrophage foam cells [225]. Mechanistically, HSPB1 promotes ABCA1 transporter activity via the PI3K/PKCζ/Sp1 signaling pathway, while HSPA1 paradoxically promotes cholesterol efflux by downregulating ABCA1/ABCG1 activity through JNK/Elk-1 signaling [224,226].

Regarding LD lipophagy/lipolysis, the mechanism is functionally dependent on the LD surface protein perilipin (PLIN) 2 (PLIN2)/PLIN3 and the lysosomal membrane receptor LAMP-2A interaction [227]. However, the facilitation of this receptor-surface protein interaction is Hsc70 mediated, as indicated by the increased colocalization of Hsc70 with PLIN2/3 levels in liver cells during starvation [227]. Expectedly, LAMP-2A deficient cells and/or defective Hsc70 activity demonstrably block their essential role in lipophagy. Moreover, HSP70 is recruited to the LD monolayer membrane through non-covalent interactions, where it chaperones nascent/denatured LD surface proteins [228]. Exploiting this system, viruses like porcine reproductive and respiratory syndrome virus 2 (PRRSV2) mechanistically mediate their replication, essentially through a Rab18/HSPA8/PLIN2 complex and a CMA-mediated lipolysis pathway [229].

However, the specific mechanisms of LD-associated HSP activities are context-dependent and influenced by the cellular metabolic landscape. While HSP70 can stabilize FSP27, its activity might exert opposing effects under varying conditions. A significant illustration is HSP70 downregulation of ABCA1/ABCG1 receptor expression through JNK/Elk-1 signaling in atherosclerosis, indicating possible context effects on cholesterol efflux [226].

Furthermore, HSP function is profoundly influenced by cellular metabolism. A notable case is reported by Weng and colleagues, where HSP60, a mitochondrial chaperone, influences electron transport chain function and fatty acid oxidation (FAO), and lipolysis through LD biogenesis [230]. As expected, HSP60 knockdown impairs FAO via SIRT3 signaling, compromising AMPK and peroxisome proliferator-activated receptor α (PPARα)-regulated FAO markers [230,231], highlighting the integration of mitochondrial chaperones with LD-associated metabolic pathways. Adipocytes upregulate the expression of HSP27, HSP60, HSP70, HSP90, and Grp78 in response to heat stress [231], suggesting a broader cellular response that impacts LD metabolism and activities.

The connection of activities of the HSPs within a signaling network, instead of isolated functions, dictates the cellular lipid phenotype. Elucidating these intricate dependencies is paramount to fully understanding the role of the chaperome in lipid balance and for translational application.

## 10. Cancer Cell Membrane Remodeling: Stress Before Membrane-Bound Epichaperome, or Vice Versa?

Cancer initiation and progression are sculpted through profound signalome/interactome remodeling that drives novel, irregular molecular interactions [232,233]. This complex rewiring promotes the emergence of unprecedented cellular behaviors and maladaptive phenotypes that define the malignant state. A part of this transformative remodeling includes changes to the PM lipid composition, architecture, and conformation [234,235]. These changes reflect a reduction in polyunsaturated fatty acid (PUFA) content within specific phosphatidylserine and phosphatidylethanolamine species, favoring a more ordered and rigid membrane domain [235,236,237,238]. Broad alterations in PM lipid composition, often featuring increased cholesterol, sphingomyelin, glycosphingolipids, and hexosylceramides, and an increase in exoplasmic content of phosphatidylserine, are consistently reported across tumor types, including breast, prostate, glioblastoma, and colorectal cancers, although the magnitudes may vary. Cancer cells specifically alter key PM lipids—including cholesterol, sphingolipids, and phosphatidylinositol 4,5-bisphosphate—and modulate the activity of enzymes that reshape the PM’s physicochemical properties contingent on the malignant cell phenotype [15,239,240,241].

The HSPs, beyond stress responders, are active architects of the plasma membrane, possessing control over its structure and function [205], a discovery that has redefined our understanding of membrane biology in ways previously unimagined over the past decade. However, it remains a challenging question: does membrane remodeling drive HSP recruitment and activity at the PM or vice versa, as accumulating literature is discovering? This raises a classic chicken-and-egg question—does membrane remodeling recruit HSPs, or do HSPs drive the remodeling? The exact details are likely more nuanced. However, it conceals a more detailed and nuanced reality. Current evidence suggests a feedback loop. Resolving the directionality will require acute, orthogonal perturbations to disentangle cause and effect.

In this respect, rather than viewing this as a linear sequence, it will be more productive to consider the interplay between the PM remodeling and HSP activity as a feedback loop. Emerging evidence suggests that both scenarios occur, with each influencing the other to shape the cellular landscape of cancer. PM lipid composition has a direct effect on protein localization and activity. Perturbing these lipids, even without direct cellular stress, can influence HSP recruitment.

However, HSPs do not just play a passive role by just responding to PM changes. They function as active remodelers, directly interacting with PM lipids and enzymes, influencing the membrane properties. HSP17, αβ-crystallin, and HSPB1/HSP27 exemplify the remodeling capability of HSPs. HSP17 and αβ-crystallin interact with the PM hydrophobic core in the lipid crystalline phase, preserving elevated lipid order and preventing destabilizing inverted hexagonal structure formation. The effect here is membrane stabilization, particularly against heat-induced hyperfluidity in specific PM microdomains (monogalactosyldiacylglycerol and phosphatidylglycerol and unsaturated lipid content), which is absent in HSP17-mutant *Synechocystis* strains [50,90]. HSPB1/HSP27 also interacts with the PM but targets distinct microdomains different from HSPB5/αβ-crystallin and HSP17 [52,68]. This reveals the intricate way in which HSPs modulate PM stabilization of the cell and the influence of the lipid composition on these processes. Also, HSP70 and DnaK (bacterial HSP70) have been hypothesized to spontaneously interact with phospholipids and insert into the hydrophobic core and stabilize the plasma membrane, as reviewed here [72,242]. These observations and hypotheses suggest a remodeling and protective role of HSPs in physiological and stress-induced conditions. Its relevance in mammalian PM remains to be confirmed.

Furthermore, insights into GRP94, a key component of the epichaperome, suggest a possible epichaperome PM modulation. With 6 putative N-glycan acceptor sites, aberrant N-glycosylation targets GRP94 and alters its conformational fitness and stability. This, in turn, may drive a sustained HSP90-mediated epichaperome interactome at the PM that promotes novel cancer phenotypes [243]. However, studies of a direct epichaperome impact on the PM remain unexplored. Nonetheless, indirect effects remain highly likely in orchestrating the unique signaling landscape of the cancer cells.

**Figure 4:**
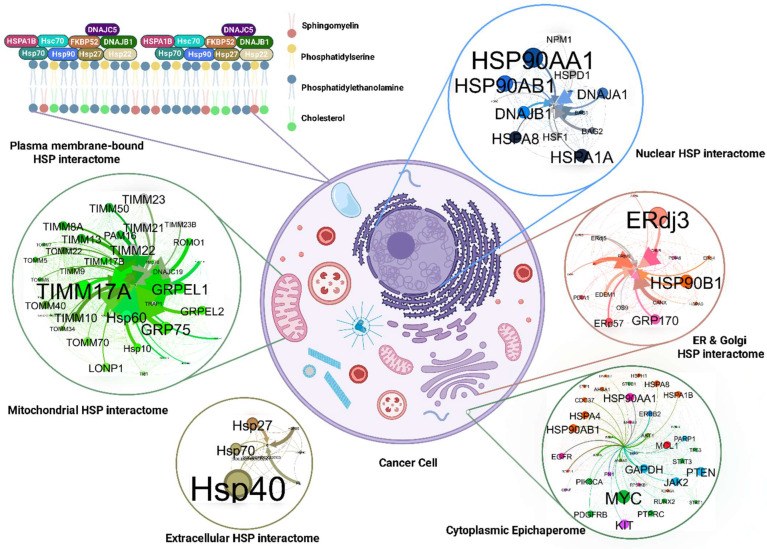
This figure visualizes the diverse and interconnected HSP interactome within a cancer cell, illustrating the specific HSPs in different cellular compartments. The pleiotropic roles of these chaperones in regulating cellular stress responses, membrane remodeling, signal transduction, and ultimately, cancer progression. The figure underscores the need for targeting specific HSP interactomes to selectively disrupt cancer cell survival and adaptation.

Additionally, our group [40,54,63,107] and others [243,244,245] have suggested the possibility of epichaperome-like interactome existence in different cellular compartments. The PM-bound epichaperome mediates novel cellular traits by integrating with diverse intracellular networks, moving beyond linear cause-and-effect as indicated in Figure 4. Selective pressure favors the translocation and activity of a specific, membrane-bound epichaperome before full transformation. While stress amplifies these changes, this pre-existing epichaperome primes the plasma membrane for unique cellular behaviors.

To fully grasp the role of epichaperome in cancer, the definition of tumor microenvironment stress should be expanded beyond heat and hypoxia. Nutrient deprivation, pH alterations, immune cell interactions, and the accumulation of metabolites, all of which contribute to this complex landscape, cannot be overlooked. The PM epichaperome, at the forefront of stress sensing, warrants extensive investigations to elucidate its diverse response to these factors.

## 11. Circadian Dynamics of Lipids and Epichaperome

Circadian rhythm, the master regulator of cellular homeostasis, modulates the circuitry of systems in anticipation of periodic oscillations and environmental stimuli. These include light–darkness cycles, sleep–wake patterns, nutrient availability and metabolites, and temperature fluctuations, which activate diverse cellular responses [246,247,248,249,250]. Coordinated, in part, by *Rev-erb-α*/*β*, the circadian clock governs fundamental cellular functions, including stress responses and metabolism, through core circadian genes like *Per*, *Cry*, and *Clock*:*Bmal1* [251,252]. This crosstalk between the circadian clock, protein activity [253], and lipid metabolism [254] positions fluctuations in lipid metabolism, its metabolites, and the expression of stress-elicited HSF and HSP genes as important regulators of the PM. Bringing us to the central question of how circadian rhythm, working through HSP, and lipid metabolism impact cellular function.

The circadian rhythm, governing gene circuitry activities, orchestrates a harmonious triad with HSPs and lipid metabolism to build and maintain cellular integrity. This system manifests in the proper expression and activity of crucial lipid-metabolizing enzymes, including pancreatic lipase, *LPLAT*, *PAP1*, *HSL*, *ACECS1*, *CYP7A1*, *HMG-CoA* reductase, and *MTTP* [255,256]. *Rev-erb-α*, for instance, regulates lipid metabolism through digestion and absorption by regulating bile salt secretion and SREBP signaling [257,258]. The HSP expression regulator, HSF, is also tightly regulated by the circadian rhythm, further emphasizing the system’s interconnectedness. The regulatory circuitry extends to a reciprocal feedback loop, as demonstrated by Necdin-mediated regulation of Bmal1 levels through SGT1-HSP90 (HSP90AA1 and HSP90AB1) interaction [259]. Likewise, HSP90 stabilizes ZEITLUPE (ZTL), an Arabidopsis circadian clock-associated F-box protein [259,260]. Further, Hsf1 interacting with *Bma1:Clock*, together with *Per2* expression, synchronizes the HSR and circadian thermal stress conditions [254,259]. HSP27 and HSP70 expression, under the control of lipogenesis, modulates *PPARγ*, *C/EBPα*, *SCD*, and *FAS* mRNA expression in adipocytes [261]. Again, correlational relationships between HSP90 activity and *PPARγ*, *Fas*, and *SREBP-1c* mRNA and protein levels have also been reported [262], further underscoring the complex relationship between the triad.

However, the interruption of the circadian triad circuitry creates a discordant system [253,263,264]. This dysregulation affects the expression and activity of lipid-metabolizing enzymes, disrupting lipid homeostasis. A case in point highlights the downregulation of *SCD* and *LPLAT* expression that alters plasma membrane lipid saturation and triggers unfolded protein response, activating GRP78 expression [265]. In another illustrative study, using melanoma cells, diacylglycerol kinase α (DGKα) and HSP27 interaction affects its activity in catalyzing saturated and monounsaturated fatty acids (SFA/MUFA)-containing phosphatidic acid (PA). Intriguingly, HSP27 and DGKα have been noted to colocalize in a DGKα-dependent fashion [266]. The resulting dynamics of monoacylglycerols and free fatty acid levels significantly affect sphingo- and phospholipid biogenesis, profoundly impacting PM properties. High lipid levels have been shown to induce HSP70 overexpression, enhancing increased cholesterol and triglyceride synthesis via *FASN*, *SCD*, and *ACC* expression [202], further underscoring a crucial bidirectional relationship between lipid metabolism and cellular stress response. Additionally, the circadian circuitry controls glycerophospholipid, cholesterol, and sphingolipid homeostasis [267,268].

Dysregulation within this circuitry disrupts lipid-handling enzymes and compromises PM function, as exemplified by HSP70 overexpression. This cascade impairs lipid homeostasis, compromises membrane integrity, and promotes intracellular and membrane lipid accumulation, thereby increasing cellular vulnerability and driving disease pathogenesis [269].

Despite the advances made so far, key questions remain unanswered. What specific lipid alterations, stemming from circadian dysregulation, compromise PM integrity? Also, how does the circadian clock influence the epichaperome and its interactions with the plasma membrane? Addressing these questions is crucial for a more comprehensive view of the role of the circadian rhythm in lipid metabolism and membrane function.

Advancing trends demand the integration of lipidomics with chaperome studies. This combined approach could unlock deeper understanding of circadian rhythms, lipid metabolism, and HSP trajectories.

## 12. Lipidomic Approaches to Studying Epichaperome–Plasma Membrane Lipid Dynamics

Heat shock proteins modulate cellular functions and respond to perturbations by forging new PPIs [107] and modulating the PM properties and membrane protein activities [52,68]. These intricate, dynamic, and transient interactions between HSPs and their interactome regulate lipid organization to modulate cell membrane changes that culminate in adaptive cellular states. Accordingly, understanding this intricate relationship is paramount to deciphering physiological and disease pathogenesis, wherein lies the potential to identify novel therapeutic targets. However, capturing and characterizing these interactions in a native cellular environment remains a challenge. The limitations of traditional methods lie in the approaches to sample preparation, their inability to preserve the delicate PM spatiotemporal pattern of events, and the narrow range of lipid species analysis [270]. This creates a gap that leaves many critical questions unanswered.

Therefore, the complexity of these interactions necessitates advanced tools to trace real-time PM changes and spatiotemporal localization of membrane–protein interactions. Fortunately, cutting-edge techniques like lipid nanodiscs and freestanding bilayers to advanced spectroscopic, proteomic, lipidomic, and computational methods offer revolutionary solutions.

Lipid nanodiscs, nanoscale phospholipid bilayers stabilized by membrane scaffold protein, offer a near-native alternative to detergent-based methods, overcoming phospholipid solubility difficulties and providing a platform to study these interactions in the complexity of the full cell membrane. For instance, a phosphatidylethanolamine-nanodisc enriched with phosphatidic acid (PEPA-nanodisc) system has successfully captured and characterized Caj1, an HSP40/J-protein with membrane protein quality control functions, exhibiting membrane affinity and phosphatidic acid specificity [271].

Yet another complementary method worth considering is freestanding lipid bilayer studies, which offer a simplified, near-native cell membrane model. Combining these with single-particle tracking allows for the observation of HSPs within phase-separated regions, alongside lipid-protein and PPIs and membrane dynamics [272]. The simultaneous characterization of ion channel open probability further enhances the comprehensive view of membrane function.

Revolutionary spectroscopic methods such as anisotropy, vibrational Raman spectroscopy, and single-molecule FRET hold promise to unveil crucial molecular events. With computational proteomics, these techniques can detect and characterize membrane-bound epichaperomes within microdomains and reveal their membrane modulation impact. Moreover, the integration of single-molecule fluorescence, neutron scattering, and all-atom molecular simulations provides a comprehensive tool for studying the molecule-spanning conformations of HSP90. Applying these to plasma membrane HSPs could clarify how localized membrane fluctuations such as membrane fluidity, viscosity, lateral pressure, thickness, hydration, and phase separation trigger global conformational shifts, thereby unraveling critical regulatory mechanisms.

Additionally, spectral phasor analysis of LAURDAN fluorophore probes, readily captured with hyperspectral imaging on confocal microscopes, allows for the resolution of PM packing, disordered-phase, and hydration dynamics [273,274]. Merging spectral phasor analysis with total internal reflection-correlation fluorescence correlation spectroscopy (TIR-FCS) [275,276] and solvatochromic probes [277,278,279] can provide subtle mechanistic observations into PM properties and interactions, revealing responses to perturbations, signaling, and the evolutionary trial of cellular transitions. By quantifying real-time changes in lipid order and hydration, these techniques could potentially shed light on early cellular transformations and membrane interactomes.

Finally, leveraging proximity-labeling lipid identification with mass spectrometry (PL-LipID-MS) and photo-crosslinkable lipid probes combined with co-immunoprecipitation and mass spectrometry (CLIP-Lipid-CoIP-MS) could offer a comprehensive exploratory means to observe real-time global lipid composition changes and epichaperome localization. High-resolution spatial resolution analysis of lipid profiles can contribute significantly to resolving the question of whether membrane remodeling drives HSP recruitment and activity at the PM or vice versa or a combined effect of both. In addition, the application of biorthogonal fluorescence reporter tools holds promise for identifying actionable targets for translational applications in PM-HSP research [280].

The synergistic combination of experimental data with computational simulations is essential to bridging scales, facilitating predictions, validating mechanisms, and illuminating these complex membrane processes. Developing systems-level analysis algorithms and models for epichaperome–lipid interactions will undeniably also provide a robust framework for extending our understanding. Collectively, these advanced techniques hold the key to comprehending the dynamic synergy between membrane lipids and the epichaperome, paving the way for significant advancements in our understanding of cellular biology and disease.

## 13. Targeting Lipids and HSPs in the Cancer Cell Plasma Membrane

Cancer multi-drug resistance (MDR) has ruined the significant progress made over the past decades in drug development, prompting the exploration of novel therapeutic strategies. In this light, membrane-lipid therapy (MLT) has emerged as a promising approach by directly manipulating PM architecture and cellular activity. MLTs encompass a diverse range of strategies, broadly categorized by their mechanism of action, including lipid-based drug delivery systems, immunotherapies, and direct modulators of membrane structure and composition. These MLTs operate through diverse molecular mechanisms, including direct membrane structure modification (type 1), regulation of PM lipid-enzymes (type 2), modulation of lipid composition-associated genes (type 3), alteration of membrane lipids to regulate PPI in specific microdomains (type 4), and modification of membrane-binding proteins (type 5) [281].

Several studies highlight the potential of targeting specific membrane lipids to disrupt cancer cell function. The novel anticancer drug hydroxytriolein (HTO), for instance, has been reported to disrupt the PM structure by impacting ordered and disordered membrane regions in TNBC [282]. This disruption, mediated by alterations in triacylglycerol composition, influences Akt signaling and dihydroceramide production, ultimately inhibiting TNBC cell proliferation and viability. Furthermore, inhibiting key enzymes involved in lipid metabolism, such as serine palmitoyltransferase (SPT) with myriocin, phosphoglycerate dehydrogenase (PHDGH) [283], PS synthesis (PTDSS1 synthase inhibitor) [284], ACAT activity (Nevanimibe, Sandoz 58-035, and AZD 3988) [285,286], or SMS enzymes (D609) [287], disrupts membrane lipid composition and sensitizes tumors to metabolic stress. These findings underscore the critical role of lipid metabolism in the ups and downs of cancer cell survival and the potential of targeting these pathways.

Lipid-based drug delivery systems, particularly liposomes, offer another innovative alternative to enhance drug delivery to overcome bioavailability challenges and modulate PM fluidity. Liposomal encapsulation, with 15% 2-OHOA, a synthetic oleic acid derivative, has come up as a practical approach to improving drug delivery [288]. Mechanistically, 2-OHOA disrupts the PM lipid conformation by increasing sphingomyelin content by controlling sphingomyelin synthase activity and impacting PKC/Ras protein membrane translocation and CDK inhibitor protein overexpression, ultimately inhibiting MAPK signaling and inducing apoptosis [289,290]. Parsa et al. also demonstrated that liposomes co-encapsulating cisplatin and doxorubicin improved targeted delivery, reduced systemic toxicity, and altered PM fluidity, leading to enhanced intracellular drug concentrations and therapeutic efficacy. Cationic liposomes further enhance drug uptake and alter PM lipid properties, impacting membrane fluidity, receptor-mediated endocytosis, and related membrane trafficking [291,292].

Additionally, the HSP co-inducer BGP-15, a type 5 ML, enhances cytoprotection through epigenetic modulation [293] and PM remodeling [294]. BGP-15 acts by inhibiting deacetylases (HDACs), increasing histone acetylation and chromatin accessibility, thereby priming heat shock genes for transcription [293]. Concurrently, BGP-15 remodels lipid raft microdomains to initiate stress signaling that culminates in HSP expression, a process that likely involves HSF1. This dual mechanism underpins the potential of BGP-15 to bolster cellular resilience and opens possibilities for broader applications of MLT, such as cardiac cardiomyocyte repair and HSP-targeted cancer therapies.

Hence, clinically, targeting PS with bavituximab plus pembrolizumab has shown promise in hepatocellular carcinoma [295], highlighting the therapeutic potential of modulating membrane lipid conformational changes. While MLTs hold significant promise, key challenges remain in achieving specificity and overcoming drug resistance, as cancer cells can adapt to their lipid metabolism. Nanoparticle characteristics, including lipid composition, size, charge, and surface properties, critically influence biodistribution, cellular uptake, and controlled drug release. Engineering these characteristics is crucial to optimize therapeutic outcomes and minimize off-target effects. Overcoming these limitations through targeted delivery strategies, combination therapies, and a deeper understanding of cancer cell lipid metabolism is crucial for the successful clinical translation of MLT-based therapies, paving the way for more effective and personalized cancer treatments.

## 14. Discussions, Perspectives, and Future Directions

Cellular homeostasis, particularly under conditions of proteotoxic stress, depends on the dynamic partnership between HSPs and the composition and speciation of membrane lipids—a collaboration that shapes the PM and influences cell fate. These molecular chaperones, long viewed as mere stress responders, are now recognized as active architects of the cellular landscape [52,68]. This complex connection involves HSP-mediated modulation of lipid biosynthetic enzymes, impacting PM lipid composition and subsequently membrane trafficking and associated signaling cascades. In cancer, this collaboration takes an anomalous turn, as tumor cells exploit the HSP–lipid axis to remodel their PM to evade apoptosis and develop multidrug resistance [243]. This tumorigenic remodeling, characterized by altered lipid asymmetry and microdomain reorganization, presents both a challenge and an opportunity for therapeutic intervention.

The paradigm shift requires acknowledging the PM as a dynamic signaling platform where lipids and proteins converge to regulate cellular processes. Precise HSP localization within the PM, critical for their function, may be heavily influenced by lipid translocases like flippases, scramblases, and floppases [296]. These enzymes maintain lipid asymmetry, which can impact the recruitment and stabilization of HSPs at specific membrane microdomains. Furthermore, alterations in lipid translocase activity may contribute to the increased PM presence of HSPs observed under stress and in oncogenesis. Moreover, disruptions in circadian rhythm-regulated lipid metabolism [246,247,248,249] and the recognition of membrane-bound epichaperomes—multiprotein complexes modulating chaperone activity—[40,54,63,107] further highlight this complexity.

The future lies in targeting the entire interconnected HSP–lipid network, not just individual components. This necessitates integrating advanced lipidomic and proteomic analyses with high-resolution imaging techniques and computational modeling to dissect the molecular mechanisms underpinning this synergy. We must leverage MLTs and combine them with HSP modulators to overcome drug resistance [281] and enhance immunotherapy application. Further, considering the established role of the *Myc* gene as a key promoter of cytoplasmic epichaperome formation [85], studies should investigate its potential involvement in chaperone networks at the plasma membrane.

Future research should focus on key areas: elucidating the mechanistic pathways governing HPS-lipid interactions, exploring the influence of circadian rhythm on this axis, and developing strategies for targeting epichaperomes. Understanding the role of flippases, scramblases, and floppases in regulating HSP localization at the PM is equally paramount. This includes determining how changes in translocase activity drive increased PM HSP presence during stress and in cancer. High-resolution structural biology will also be crucial for mapping protein-lipid interactions (PLIs) and protein–protein interactions (PPIs) within the PM. Advanced imaging techniques, such as super-resolution microscopy and Förster resonance energy transfer (FRET), will enable real-time visualization of these dynamics. Systems biology approaches, integrating multi-omics data, will also allow proactive modeling of the complex HSP–lipid network [270]. Therapeutically, MLT strategy optimization must develop inhibitors targeting specific HSP isoforms and engineer targeted nanoparticles for precise drug delivery to PM microdomains [288].

Beyond conventional approaches, concepts such as designing artificial epichaperomes with tailored functions to modulate cellular behavior, harnessing HSPs to promote regenerative processes by manipulating membrane structure, and investigating the role of membrane tension in regulating HSP activity and lipid organization offer unexplored territories. By pursuing these ambitious directions, we can unlock the full therapeutic potential of the HSP–lipid axis, providing innovative strategies to combat disease and understand fundamental cell biology.

To reiterate our intent, this review presents a comprehensive analysis of HSP–lipid interactions yet is selectively detailed in less researched related areas. We synthesize current understanding of HSP–lipid domain organization, identify key knowledge deficits—particularly related to in situ validation, therapeutic strategies, and biomarkers—and outline critical directions for future investigations.

**Outstanding questions**
1. Does acute, selective depletion of membrane epichaperome components (degron or rapid pharmacology) change PM order and signaling as measured by live-cell LAURDAN imaging?2. What are the direct lipid species partners of the membrane epichaperome in native PMs?3. Could native single-channel recordings confirm physiologically relevant HSP-formed conductances?4. Do in vivo epichaperome perturbations reshape membrane lipidomics and trafficking?


## 15. Conclusions

The plasma membrane, with recent advancements in lipidomics and proteomics, has unveiled a more sophisticated and dynamic structure of its widely accepted fluid mosaic model. Within the continuum of this structural framework lie plasma proteins, including the molecular chaperones. This paper proposes that the continuum of chaperones at the PM forms an “epichaperome–plasma membrane lipid axis.” We hypothesize that chaperones within this axis integrate to create a functional plasma membrane epichaperome. This membrane-bound epichaperome, with its characteristic functional domains, likely orchestrates de novo cell adaptive plasticity, particularly in response to exogenous stress. In the context of oncogenesis, the epichaperome–plasma membrane lipid axis is co-opted and networked with epichaperome hubs in other cellular compartments. This interconnection sustains the aberrant signaling cascades and augmented survival characteristic of malignancy.

The epichaperome–plasma membrane lipid axis has the potential to profoundly propagate critical cellular processes implicated in cancer. These include endocytic internalization, exosomal vesicular exocytosis, and the formation of tunneling nanotubes. Recognizing the critical role of this axis in driving these oncogenic phenotypes is crucial. This understanding could lead to precisely targeted strategies aimed at the epichaperome–plasma membrane lipid axis. These strategies could involve modulating the supramolecular epichaperome assembly or exploiting specific microdomains to compromise the adaptive circuitry of cancer cells.

Thus, future advancements will depend on several key areas. These include high-resolution spatiotemporal mapping of lipid–chaperone molecular interactions, sophisticated artificial intelligence models that connect molecular interactions to systems-level phenomena such as altered cellular behavior and drug resistance, and experimental paradigms that meticulously incorporate contextual influences like circadian rhythms and biomechanical cues. These approaches hold great promise.

Unraveling the intricate mechanisms governing the epichaperome–plasma membrane lipid axis would require interdisciplinary collaboration. This collaboration must encompass expertise from biochemistry, biophysics, computational biology, and oncology. Ultimately, a comprehensive understanding and targeted manipulation of this axis offer the potential for developing innovative and effective therapeutic interventions against cancer.

## Figures and Tables

**Table 1:** A Summary and Categorization of HSPs Interactions with Plasma Membrane Lipids.

HSP(s)	Lipid Species/ Membrane Context	Type of Lipid Object and Experimental System	Mechanistic Insight	Category	Reference
HSP70 (HSPA1A)	Palmitoyloleoyl phosphatidylserine, tetraoleoyl cardiolipin, bis-monoacylglycerophosphate	Large unilamellar vesicles; tryptophan fluorescence spectroscopy, circular dichroism	Peripheral binding via Trp90/Trp580, insertion into hydrophobic cavities, and selective binding to specific lipid headgroups	Model membrane system	[35]
HSP90 (Hsp90AA1)	Palmitoyloleoyl phosphatidylcholine, palmitoyloleoyl phosphatidylethanolamine, palmitoyloleoyl phosphatidylserine, dioleoyl phosphatidylinositol, sphingomyelin	Small unilamellar vesicles; surface plasmon resonance (SPR) assay, far-UV circular dichroism, liposome leakage assay	Insertion into the lipid bilayer and promotes membrane stabilization	Model membrane system	[55]
HSP90	Cholesterol-rich domains	KB cells, pure lipid; immunofluorescence analysis, steady-state fluorescence anisotropy, tryptophan fluorescence	Localization in cholesterol-rich membrane microdomains facilitates cholesterol redistribution	Cellular/Animal model system	[39]
Hsp70	Palmitoyloleoyl phosphatidylserine, dipalmitoyl phosphatidylserine, dioleoyl phosphatidylserine	Liposomes; Western blot	Affinity elevation by increasing the saturation of the lipid fatty acid chain	Model membrane system	[56]
Hsc70/HSPA8	Palmitoyloleoyl phosphatidylserine, bis-dimyristoyl cardiolipin	Liposomes, isothermal titration calorimetry, Western blot	ATP/ADP-dependent Hsc70 binding and possible Hsc70 recruitment of HSP90 to anionic domains (hypothetic)	Model membrane system/Speculative model	[42]
HSP22	Phosphatidylinositol, Phosphatidylserine, Phosphatidic acid	Small unilamellar vesicles	Amphitropic membrane binding; stabilization of inner leaflet of anionic lipids	Model membrane system	[57]
HSPB6	1,2-dimyristoyl-sn-glycero-3-phospho-L-serin, L-α-phosphatidylcholine, L-α-phosphatidylethanolamine; L-α-phosphatidylinositol-4-phosphate; L-α-phosphatidylserine	Liposome assays	Lipid-dependent (PS/Cholesterol) chaperone activity; inhibition of α-synuclein aggregation	Model membrane system	[58]
HSP17	Dioleoyl phosphatidylcho-line, dioleoyl phosphati-dylglycerol, individual lipid classes of Synecho-cystis, thylakoid membrane	Large unilamellar vesicles, lipid monolayers, fluorescence anisotropy, FTIR	Specificity for anionic lipids, elevation of physical order of model lipid membranes	Model membrane system	[50]
Lo18 (sHSP, *Oenococcus oeni*)	Synthetic bilayer	FTIR, Calorimetry	Modulates the membrane bilayer packing	Model membrane system	[59]
Small HSP (HSPB1)	Dioleoyl phosphatidylcholine, palmitoyloleoyl phosphatidylcholine, sphingomyelin, dehydrocholesterol, B16-F10 *E. coli* cell membrane	Lipid monolayers, pure lipids, giant unilamellar vesicles, cell membrane; surface pressure analysis, EPR spectroscopy, ITIR-FCS, calculation of general polarization	Affinity towards highly fluid membranes, alterations in rotational and lateral lipid mobility	Model membrane + Cellular/Animal model system	[52]
GroEL	Dioleoyl phosphatidylglycerol, dipalmitoyl phosphatidylcholine, dimyristoyl phosphatidylcholine	Large unilamellar vesicles, lipid monolayers; SDS-PAGE, steady-state fluorescence anisotropy,	Increase in lipid order	Model membrane system	[51]
Hsp70	1-palmitoyl-2-oleoyl-phosphatidylethanolamine, and 1-palmitoyl-2-oleoyl-phosphatidylserine	Immunostaining; Detergent-resistant membrane isolation; Protein expression studies; Microscopy	Stress-induced PM translocation of Hsp70 to the extracellular membrane compartment to induce immune cell (macrophages) activation (evidenced by TNF-α production)	Model membrane + Cellular/Animal model system	[60]
HSP70	Cancer cell PM (B16-F10 and A375 cells)	AFM, Cell-based assay	Oligomerization at PM; regulates clathrin-independent endocytosis	Cellular/Animal model system	[61]
Stress-inducible Hsp70 (HSPA1A)	Palmitoyloleoyl phosphatidylserine, dipalmitoyl phosphatidylserine, dioleoyl phosphatidylserine	Liposomes; Western blot	Embedding elevation by increasing the saturation level of phosphatidylserine	Model membrane system	[36]
HSP27	Lipid raft-associated ERK1/2	Human glioblastoma (GBM) cells-derived exosomes uptake by endothelial and fibroblast cells	Exosome uptake via ERK1/2-HSP27 signaling (direct and mechanistically)	Cellular/Animal model system	[62]
HSP12 (*S. cerevisiae*)	Dimyristoyl phosphatidylglycerol	Liposomes; freeze-fracture electron microscopy, NMR spectroscopy, differential scanning calorimetry	Influence on the membrane phase segregation by forming an intermediate ripple phase; modulates bilayer order	Model membrane system	[49]
Stress-inducible Hsp70 (HSP70-1A)	Dipalmitoyl phosphatidylserine/phosphatidylcholine bilayers with/without cholesterol	Supported lipid bilayers; atomic force microscopy	Specific interaction with ordered dipalmitoyl phosphatidylserine domains to regulate the formation of protein clusters	Model membrane system	[32]
mHSP70	Lipid rafts (Glioblastoma)	Detergent lipid extraction, Cell-based assays, Live-cell imaging, Chromatography and Mass Spectrometry	PM clustering; invasion/motility regulation	Cellular/Animal model system	[63]
Hsp70	Dimyristoyl phosphatidylserine, dipalmitoyl phosphatidylserine, palmitoyloleoyl phosphatidylcholine, palmitoyloleoyl phosphatidylserine, dipalmitoyl phosphatidylcholine	Giant unilamellar vesicles; differential scanning calorimetry, confocal microscopy	Influence on the thermotropic behavior of phosphatidylserine, induction of interdigitation, preference for an ordered lipid phase	Model membrane system	[54]
mHSP70	Gb3-rich lipid rafts	Methyl-cyclodextrin treatment, STED nanoscopy, Immunofluorescence; Flow cytometry	Supports tunneling nanotube formation	Cellular/Animal model system	[64]

This table systematically stratifies HSP–lipid interactions at the PM and assigns the evidence of the model systems used in the respective studies (liposomes, supported bilayers, and GUVs), cellular/animal model studies (live-cell imaging, functional assays), and speculative models.

## Data Availability

No new data were created or analyzed in this study.

## Abbreviations

The following abbreviations are used in this manuscript:

2-OHOA	2-hydroxyoleic acid
ABCA1	ATP-binding cassette transporter A1
ABCG1	ATP-binding cassette transporter G1
ACAT	Acyl-CoA: Cholesterol Transferase
ACC1	Acetyl-CoA carboxylase 1
ACECS1	Cytoplasmic acetyl-CoA synthase
AMPKα	AMP-activated protein kinase
CFTR	Cystic fibrosis transmembrane conductance regulator
COPII	Coated protein II
CSPs	Cysteine string proteins
CYP7A1	Cholesterol-7 alpha hydroxylase
EEA1	Early endosome effector protein
FASN	Fatty acid synthase
FKBP8	38 kDa FK506-binding protein
HGGCS1	3-hydroxy-3-methylglutaryl-CoA synthase 1
HMG-CoA reductase	Beta-hydroxy beta-methylglutaryl-coenzyme A reductase
HMGCR	HMG-CoA reductase
HSL	Choline kinase, hormone-sensitive lipase,
HSR	Heat Shock Response
INSIG	Insulin-induced gene
LDAF1	Lipid droplet assembly factor 1
LDLR	Low-density lipoprotein receptor
LPCAT1	Lysophosphatidylcholine acyltransferase 1
LPLAT	Lysophospholipid acyltransferase
MLT	Membrane-lipid therapy
MTTP	Microsomal triglyceride transfer protein
MUFA	Monounsaturated fatty acids
PAP1	Phosphatidate phosphohydrolase 1
PPARγ	Peroxisome proliferator-activated receptor γ
PTDSS1	Phosphatidylserine synthase inhibitor,
Scd1	Stearoyl-CoA desaturase 1
SFA	Saturated fatty acids
SMS	Sphingomyelin synthase
SNARE	Soluble N-ethylmaleimide-sensitive factor attachment protein receptor
TNBC	Triple Negative Breast Cancer
TNFAIP2	Tumor necrotic factor-α-induced protein 2
VAMP	Vesicle-associated membrane protein
